# Evaluating the association of biallelic *OGDHL* variants with significant phenotypic heterogeneity

**DOI:** 10.1186/s13073-023-01258-4

**Published:** 2023-11-29

**Authors:** Sheng-Jia Lin, Barbara Vona, Tracy Lau, Kevin Huang, Maha S. Zaki, Huda Shujaa Aldeen, Ehsan Ghayoor Karimiani, Clarissa Rocca, Mahmoud M. Noureldeen, Ahmed K. Saad, Cassidy Petree, Tobias Bartolomaeus, Rami Abou Jamra, Giovanni Zifarelli, Aditi Gotkhindikar, Ingrid M. Wentzensen, Mingjuan Liao, Emalyn Elise Cork, Pratishtha Varshney, Narges Hashemi, Mohammad Hasan Mohammadi, Aboulfazl Rad, Juanita Neira, Mehran Beiraghi Toosi, Cordula Knopp, Ingo Kurth, Thomas D. Challman, Rebecca Smith, Asmahan Abdalla, Thomas Haaf, Mohnish Suri, Manali Joshi, Wendy K. Chung, Andres Moreno-De-Luca, Henry Houlden, Reza Maroofian, Gaurav K. Varshney

**Affiliations:** 1 Genes & Human Disease Research Program, Oklahoma Medical Research Foundation, Oklahoma City, OK 73104 USA; 2 Institute of Human Genetics, Julius Maximilians University Würzburg, Würzburg, Germany; 3 Institute of Human Genetics, University Medical Center Göttingen, Göttingen, Germany; 4 Institute for Auditory Neuroscience and InnerEarLab, University Medical Center Göttingen, Göttingen, Germany; 5 Department of Otolaryngology-Head and Neck Surgery, Tübingen Hearing Research Center, Eberhard Karls University, Tübingen, 72076 Germany; 6 Department of Neuromuscular Disorders, Queen Square Institute of Neurology, University College London, London, UK; 7 Clinical Genetics Department, Human Genetics and Genome Research Institute, National Research Centre, Cairo, Egypt; 8 Molecular and Clinical Sciences Institute, St. George’s, University of London, Cranmer Terrace London, London, UK; 9 Department of Pediatrics, Faculty of Medicine, Beni-Suef University, Beni-Suef, Egypt; 10 Medical Molecular Genetics Department, Human Genetics and Genome Research Institute, National Research Centre, Cairo, Egypt; 11 Institute of Human Genetics, University of Leipzig Medical Center, Leipzig, Germany; 12 Centogene GmbH, Rostock, Germany; 13 Bioinformatics Centre, S. P. Pune University, Pune, India; 14 GeneDx, Gaithersburg, MD 20877 USA; 15 Department of Genetics and Genomic Sciences, Icahn School of Medicine at Mount Sinai, New York, NY USA; 16 Department of Pediatrics, Icahn School of Medicine at Mount Sinai, New York, NY USA; 17 Department of Pediatrics, School of Medicine, Mashhad University of Medical Sciences, Mashhad, Iran; 18 Department of Pediatrics, Zabol University of Medical Sciences, Zabol, Iran; 19 Department of Human Genetics, Emory University, Atlanta, GA 30322 USA; 20 Institute for Human Genetics and Genomic Medicine, RWTH Aachen University, Pauwelsstr. 30, Aachen, 52074 Germany; 21 Autism & Developmental Medicine Institute, Geisinger, Lewisburg, PA USA; 22 Department of Pediatric Endocrinology, Gaafar Ibn Auf Children’s Tertiary Hospital, Khartoum, Sudan; 23 Nottingham Clinical Genetics Service, Nottingham University Hospitals NHS Trust, Nottingham, UK; 24 Department of Pediatrics, Boston Children’s Hospitaland, Harvard Medical School , Boston, MA USA; 25 Department of Diagnostic Radiology, Kingston Health Sciences Centre, Queen’s University, Kingston, ON Canada

**Keywords:** 2-oxo acid dehydrogenase, OGDHL, Genetic compensation, Disease model, Zebrafish, Neurodevelopmental disorders, Mitochondria, Variant testing

## Abstract

**Background:**

Biallelic variants in *OGDHL*, encoding part of the α-ketoglutarate dehydrogenase complex, have been associated with highly heterogeneous neurological and neurodevelopmental disorders. However, the validity of this association remains to be confirmed. A second *OGDHL* patient cohort was recruited to carefully assess the gene-disease relationship.

**Methods:**

Using an unbiased genotype-first approach, we screened large, multiethnic aggregated sequencing datasets worldwide for biallelic *OGDHL* variants. We used CRISPR/Cas9 to generate zebrafish knockouts of *ogdhl*, *ogdh* paralogs, and *dhtkd1* to investigate functional relationships and impact during development. Functional complementation with patient variant transcripts was conducted to systematically assess protein functionality as a readout for pathogenicity.

**Results:**

A cohort of 14 individuals from 12 unrelated families exhibited highly variable clinical phenotypes, with the majority of them presenting at least one additional variant, potentially accounting for a blended phenotype and complicating phenotypic understanding. We also uncovered extreme clinical heterogeneity and high allele frequencies, occasionally incompatible with a fully penetrant recessive disorder. Human cDNA of previously described and new variants were tested in an *ogdhl* zebrafish knockout model, adding functional evidence for variant reclassification. We disclosed evidence of hypomorphic alleles as well as a loss-of-function variant without deleterious effects in zebrafish variant testing also showing discordant familial segregation, challenging the relationship of *OGDHL* as a conventional Mendelian gene. Going further, we uncovered evidence for a complex compensatory relationship among OGDH, OGDHL, and DHTKD1 isoenzymes that are associated with neurodevelopmental disorders and exhibit complex transcriptional compensation patterns with partial functional redundancy.

**Conclusions:**

Based on the results of genetic, clinical, and functional studies, we formed three hypotheses in which to frame observations: biallelic *OGDHL* variants lead to a highly variable monogenic disorder, variants in *OGDHL* are following a complex pattern of inheritance, or they may not be causative at all. Our study further highlights the continuing challenges of assessing the validity of reported disease-gene associations and effects of variants identified in these genes. This is particularly more complicated in making genetic diagnoses based on identification of variants in genes presenting a highly heterogenous phenotype such as “OGDHL-related disorders”.

**Supplementary Information:**

The online version contains supplementary material available at 10.1186/s13073-023-01258-4.

## Background

The tricarboxylic acid (TCA) cycle, also known as the Krebs cycle, is a vital process for energy production and biosynthesis in higher animals, including humans. Genetic variants in enzymes constituting the TCA cycle have been associated with autosomal recessive disorders of mitochondrial metabolism. These variants can lead to inborn errors of metabolism, resulting in diverse phenotypes such as early infantile Leigh-like encephalopathy, developmental delay/intellectual disability, movement disorders, liver diseases, cardiomyopathy, and hearing and vision impairments [1–4]. Deficiency of the TCA cycle component 2-oxoglutarate dehydrogenase complex (OGDHC) causes rare autosomal recessive disease. The complex converts 2-oxoglutarate (alpha-ketoglutarate) to succinyl-CoA and consists of three enzyme subunits encoded by the 2-oxoglutarate dehydrogenase (*OGDH*) (E1; OMIM: 613022), *DLST* (E2; OMIM: 126063), and *DLD* (E3; OMIM: 238331) genes [5]. The E1 component of OGDHC is encoded by *OGDH*. Biallelic pathogenic variants in this gene are associated with a neurodevelopmental disorder characterized by global developmental delay, movement disorder, and metabolic abnormalities (OMIM: 203740) [6]. In humans, mice, and zebrafish, OGDH has a paralog called OGDH-like (*OGDHL*; OMIM: 617513), while *Drosophila* only possesses a single *dOgdh* gene.

OGDHL is a protein predominantly expressed in the brain [7], bearing similarities to OGDH. The precise functional role of OGDHL has yet to be fully elucidated. OGDH and OGDHL are involved in the metabolism of 2-oxoglutarate, an important intermediate in several metabolic pathways [8]. OGDH is the main enzyme responsible for this metabolism, while OGDHL may play a supporting role. OGDHL may be involved in the brain-specific control of 2-oxoglutarate distribution, which is an important intermediate in several metabolic pathways. 2-Oxoglutarate can be used for energy production or synthesizing the neurotransmitter glutamate, an essential neurotransmitter in various brain functions, including excitatory signaling and synaptic plasticity [7]. OGDH is more promiscuous and can accept both 2-oxoglutarate and 2-oxoadipate as substrates while its paralog *DHTKD1* encoded dehydrogenase E1 and transketolase domain-containing protein 1 is more specific for 2-oxoadipate. Loss of DHTKD1 function can lead to a cytotoxic accumulation of 2-oxoadipate and OGDH can help compensate for loss of DHTKD1 function by catalyzing the oxidation of 2-oxoadipate [9]. However, whether this functional redundancy extends to OGDHL and its clinical relevance in individuals with *OGDHL* variants remains uncertain. Biallelic *DHTKD1* (OMIM: 614984) variants lead to alpha-aminoadipic and alpha-ketoadipic aciduria, which is an autosomal recessive inborn error of lysine, hydroxylysine, and tryptophan degradation. The clinical presentation of this metabolic disorder is highly heterogenous [10]. Additionally, a segregating nonsense monoallelic *DHTKD1* variant was identified in a large Chinese family affected by Charcot-Marie-Tooth disease type 2 [11]. However, this association remains to be confirmed.

Recently, biallelic variants in *OGDHL* have been proposed to be associated with highly heterogeneous neurodevelopmental disorders manifesting with variable epilepsy, ataxia, spasticity, growth abnormalities, dysmorphism, hearing, and visual impairments [6, 12]. Several intriguing aspects of the most recent study prompted further investigation of the clinical presentation of *OGDHL* pathogenicity, necessitating further investigation into its function in a previously characterized monogenic disorder: (1) a highly heterogeneous clinical presentation clouded by the absence of a single cardinal phenotype occurring in all or most individuals or lack of biochemical biomarker; (2) the majority of the variants were missense (9/11); (3) several of the reported variants appeared with high minor allele frequency and were even present in a homozygous state in variant frequency databases; (4) the variants were identified from a relatively small cohort of 10 cases across 9 nuclear families with limited familial segregation analysis; and (5) functional studies were performed in *Drosophila* *melanogaster* lacking an OGDHL ortholog. To evaluate the potential gene-disease relationship, we collected an additional multi-ethnic cohort of 14 patients from 12 families with biallelic *OGDHL* variants to assess the potential for causality by testing all reported and new *OGDHL* variants using a newly generated zebrafish model. This model was also used to investigate *OGDHL* variant pathophysiology at the organismal and cellular levels to explore *OGDH* functional redundancy and assess its appropriateness as a functional readout of variant pathogenicity. Here, we explore the relationship between sequence, expression, and function of *OGDHL* related genes and uncovered significant genetic compensation in this metabolic pathway. Collectively, this study provides a cautionary perspective to laboratories through the assembly of a new patient cohort while unravelling complex OGDHL biology through a vertebrate model.

## Methods

### Case inclusion, genetic investigation, and clinical evaluation

As part of an effort to investigate the association of biallelic *OGDHL* variants with human disorders, we applied a genotype-first approach to screen a large set of sequencing data from different diagnostic and research genetic labs including Centogene, GeneDx, Baylor Genetics, Invitae, 3billion lab, 100,000 Genomes Project, UCL Queen Square Genomics (QSG), ClinVar, DECIPHER, DDD, Geno2MP, and many other smaller databases. Our genotype-first approach supported through international collaboration and GeneMatcher [13] identified individuals with *OGDHL* variants in different labs agnostic to specific clinical indication for detailed phenotypic analysis. This led to the identification of 14 individuals originating from UCL-QSG (*n* = 4), GeneDx (*n* = 3), Centogene (*n* = 2), as well as at three German Institutes of Human Genetics in Leipzig (*n* = 2), Würzburg (*n* = 2), and Aachen (*n* = 1). Each individual had exome sequencing applied in either a proband-only (*n* = 11) or trio-exome (*n* = 1) approach or was identified through segregation analysis following identification of an *OGDHL* variant in an affected sibling (*n* = 2). One family (family 4, with individuals 4 and 5) was subjected to genome-wide homozygosity mapping as previously described [14]. *OGDHL* variants were annotated using the RefSeq accession NM_018245.3. Variant classification and interpretation were performed following the ACMG/AMP guidelines, including the ClinGen Sequence Variant Interpretation recommendations, adapted variant of uncertain significance (VUS) categories to allow for subclassification, and applied the Varsome tool to ease the calculation of points. We applied a points system described in Tavtigian *et al*. [15] and adapted a VUS subclassification system as per Zouk *et al*. [16] and classified variants with the following points accordingly: ≥ 10 pathogenic, 6 to 9 likely pathogenic, 5 VUS-favor pathogenic (VUS-FP), 1-4 VUS, 0 VUS-favor benign (VUS-FB), -1 to -6 likely benign, and ≤ -7 benign. Functional testing of each variant in zebrafish rescue experiments rendered application of PS3_M and addition of 2 points in the event of lack of phenotype rescue and BS3_M and subtraction of 2 points in the event of phenotype rescue.

The study was approved by the institutional ethics committees of the participating centers and written informed consent was obtained from the families involved in this study, in accordance with the Declaration of Helsinki. Detailed clinical features as well as family history were obtained from all affected individuals and reviewed carefully by clinical geneticists (M.Z. and N.A.) and a neurologist (H.H.). Brain MRIs were reviewed by a board certified neuroradiologist (A.M.D.).

### Structural modelling of OGDHL variants

A structural model of OGDHL was built using the cryo-electron microscopy structure of human OGDH (PDB ID: 7WGR) [17] and the AlphaFold [18] structure of OGDHL (associated with UniprotKB ID Q9ULD0). The cryo-EM structure of OGDH (1023 aa) at 2.9 Å resolution has missing coordinates for residues between 1 to 128, 346 to 365, and 574 to 602. The AlphaFold database has a structural model for OGDHL (1010 aa), which is deposited in the monomeric state and has the highest confidence prediction levels for residues between 169 and 559 as well as 587 and 1010. The human OGDH and OGDHL proteins share 75.7% sequence identity, with variations across the length of the sequences but with conservation of the key active site and co-factor binding site residues. Homology modeling was performed with Modeler software as part of Discovery Studio 2017 version (BIOVIA, Discovery Studio Modeling Environment, Release 2019, San Diego: Dassault Systèmes). Since the patient variants of interest are not between residues 1 and 128, given the absence of EM coordinates and the lower AlphaFold confidence in this region, the structural models were built were between residues 129 and 1010. All models were built in dimeric state including TPP, Mg^2+^, and Ca^2+^. Variants were introduced by the “Mutate” option in Discovery Studio.

### Zebrafish functional studies

All experimental animal care was performed in accordance with institutional and NIH guidelines and regulations. Zebrafish (*Danio rerio*) were raised and maintained in an Association for Assessment and Accreditation of Laboratory Animal Care (AAALAC) accredited facility at the Oklahoma Medical Research Foundation (OMRF) under standard conditions, and all experiments were performed as per protocol 22-18 approved by the Institutional Animal Care Committee (IACUC) of OMRF. All zebrafish work was carried out in wild-type (WT) strain NHGRI-1 [19]. The zebrafish husbandry procedures were carried out as methods described earlier [20]. The *ogdhl* mutant was generated by CRISPR/Cas9 as described earlier [21]. The primer sequences employed for generating the *ogdhl* mutant can be found in Additional file 1: Table S1. The schematic representation of the sgRNA target sequence and the CRISPR/Cas9-induced 4 bp deletion, which led to a premature stop codon and consequent truncation of the protein are shown in Additional file 2: Fig. S1A-B.

### Generation of F_0_ knockout zebrafish

Zebrafish-specific single guide RNA (sgRNA) target sites were designed using CRISPOR tool [22] and synthesized chemically (Synthego Inc.). A mixture containing 1 μL of 40 μM Cas9-NLS protein (UC Berkeley QB3 Macrolab, Berkeley, CA), 300–500 ng of each sgRNA (in 3 μL), and 2 μL of 1 M potassium chloride was injected into one-cell stage WT embryos and phenotyping was performed in the F_0_ generation within the first 5 days post-fertilization (5 dpf). The sgRNA target sequences and PCR primer sequences are listed in Additional file 1: Table S1. Genotyping was performed as described earlier [21]. Indels were identified from a pool of embryos, PCR amplified, Sanger sequenced (Additional file 2: Fig. S1C), and analyzed by ICE analysis (Additional file 2: Fig. S1D).

### RNA extraction and real-time quantitative PCR (RT-qPCR)

Total RNA was extracted using TRIzol Reagent (Thermo Fischer Scientific, Waltham, MA, USA) and purified by miRNeasy Mini kit (Qiagen, Hilden, Germany), following the manufacturer’s instructions. For RT-qPCR, cDNA was synthesized using reverse transcription with iScript cDNA-synthesis kit (Bio-Rad, Hercules, CA, USA). The RT-qPCR reaction was done using SYBR Green super mix. The primer sequences for RT-qPCR are shown in Additional file 1: Table S1. The cycle threshold (Ct) values were imported into Microsoft Excel for relative gene expression analysis. Quantification was based on the 2^(-ΔΔCT) method using corresponding age-matched control samples as calibrators [23].

### Generation of rescue and specific variant constructs

A cDNA clone encoding for human *OGDHL* (NM_018245) was purchased from Origene Technologies, Inc. (Rockville, MD, USA). This clone harbors two rare variants (L511 and N573) and were changed back to WT amino acid P511 and D573 as per NCBI reference sequence using Quick Change II kit from Agilent Technologies, Inc. (Santa Clara, CA, USA). The human *OGDH* cDNA clone (NM_002541) was acquired from transOMIC Technologies, Inc. (Huntsville, AL, USA). *OGDHL* or *OGDH* cDNA clones served as templates to amplify fragments with *Eco*RI and *Xho*I sites, and *Cla*I and *Xho*I sites, respectively. These fragments were then cloned into pCS2 + (A kind gift by Dr. David Turner, The University of Michigan, Ann Arbor, MI, USA) vector to generate the pCS2 + hOGDHL or pCS2 + hOGDH constructs. Specific variants from affected individuals were introduced in pCS2 + hOGDHL construct using site-directed mutagenesis method. All constructs were sequenced end-to-end (Primordium Lab, CA, USA). The primer sequences for cloning or site-directed mutagenesis are listed in Additional file 1: Table S1.

### In vitro transcription of capped mRNA synthesis and microinjection

Capped mRNA was synthesized by in vitro transcription after linearizing either the WT or variant-specific plasmids by *Not*I using mMessage mMachine SP6 Transcription kit (Invitrogen, CA, USA) and then purified by RNA clean and concentrator-5 kit (Zymo research, USA). For *OGDHL* or *OGDH* rescue experiments, 200 pg mRNAs were microinjected into one-cell stage embryos together with Cas9 protein and sgRNAs. In the functional analysis of *OGDHL* variants, a single variant microinjection utilized 200 pg of mRNA, while a double variant microinjection used 100 pg for each variant mRNA (totaling 200 pg for the two variant mRNAs).

### Morphological phenotyping and imaging

The phenotype analysis of *ogdhl* knockouts was conducted on embryos at the 3 dpf stage. The animals were immobilized in 2% methylcellulose (Sigma Aldrich, St. Louis, MO, USA) for imaging, using a Nikon DS-Fi2 camera mounted on a Nikon SMZ18 stereomicroscope (Nikon, Japan). Head, eye, and body lengths were measured directly from scale-calibrated images using ImageJ software (NIH). The head length was determined by tracing a line from the snout tip to the end of the otic vesicle, eye size was measured by diameter, and body size by tracing a line from the snout tip to the end of the trunk. To calculate the values, each measurement from the injected embryos was presented as a percentage difference relative to the mean value of the uninjected embryos. Heart morphology (edema) was quantified as a percentage of total number of embryos exhibiting the phenotype.

### Whole-mount immunohistochemistry and imaging

Whole-mount Immunohistochemistry was performed on 3 dpf zebrafish embryos. In brief, embryos were fixed in 4% PFA at 4 °C overnight. The fixed embryos were washed with 0.2%Triton X-100 + 1X PBS (PBSTx) and then treated with a dehydration/rehydration cycle with 25%, 50%, 75%, and 100% methanol and processed as described earlier. The specimens were subsequently transferred into blocking buffer at 4℃ overnight and then incubated with mouse IgG2b anti-acetylated tubulin monoclonal antibody (1:250; Sigma, St. Louis, MO, USA) and Alexa Fluor AffiniPure 488 goat anti-mouse IgG (H + L; 1:500, Jackson ImmunoResearch, West Grove, PA, USA). Stained embryos were mounted in 1.5% low-melting agarose (CamBrex, Rockland, ME, USA) and imaged with a confocal microscope Zeiss LSM710. For motor axon length and diameter measurements, six embryos were used for each uninjected and Cas9-injected controls and *ogdhl* F_0_ mutants. The motor axon length (5 axons per animal) was measured by tracing a line from the dorsal tip to the ventral-end of acetylated tubulin-positive axon, as indicated in the figures. The axonal diameter (5-6 axons per animal) was measured at the same position, which is closer to the axon soma as shown in the figures.

### TUNEL assay

TUNEL assay was performed with the ApopTag Peroxidase in situ apoptosis detection kit (Millipore Sigma, St. Louis, MO, USA) and the staining procedure with minor modifications as described previously [24]. The TUNEL-positive cells were counted within eye, midbrain, hindbrain, and spinal cord as indicated in figures and were calculated in the percentage of mean value of uninjected embryos.

### Zebrafish behavior assays

All behavioral tests were conducted at room temperature. To analyze the light and sound startle response, larval movements were tracked and analyzed using either Zebrabox (Viewpoint Life Sciences, Montreal, Canada) or Noldus (Noldus Information Technology, Leesburg, VA, USA) system following previously described methods [24–26]. For the light-dark transition test, larvae at 4 dpf were individually transferred to 96-well plates. Each well contained a single larva in 175 μL of E3 buffer, sealed with paraffin film. The following day, the plate was placed in the behavior recording chamber and the EthoVision XT software was used to record and analyze locomotion activity. In brief, 5 dpf larvae were subjected to a 30-min habituation period in the dark, followed by a 10-min light-dark transition for three cycles. The distance traveled in millimeters (mm) per minute and the velocity in millimeters per second (mm/s) were recorded. The data for each minute were plotted using GraphPad Prism version 9.5 (GraphPad Software, San Diego, CA, USA).

### Statistical analysis

Each experiment was repeated three times, and sample sizes are described in each figure legend. Data are presented as mean value ± standard deviation (SD). Statistical analysis was performed using GraphPad Prism version 9.4 (GraphPad Software, San Diego, CA, USA). The significance level was set to 0.05 in all analyses. The numbers of animals and independent experimental replications along with the significance are reported in the figure legends. Data were determined to be statistically significant when *p* < 0.05 (*), *p* < 0.01 (**), *p* < 0.001 (***), and *p* < 0.0001 (****). Specified statistical tests are described in the figure legends.

## Results

### Identification of biallelic variants in *OGDHL*

**Fig. 1:**
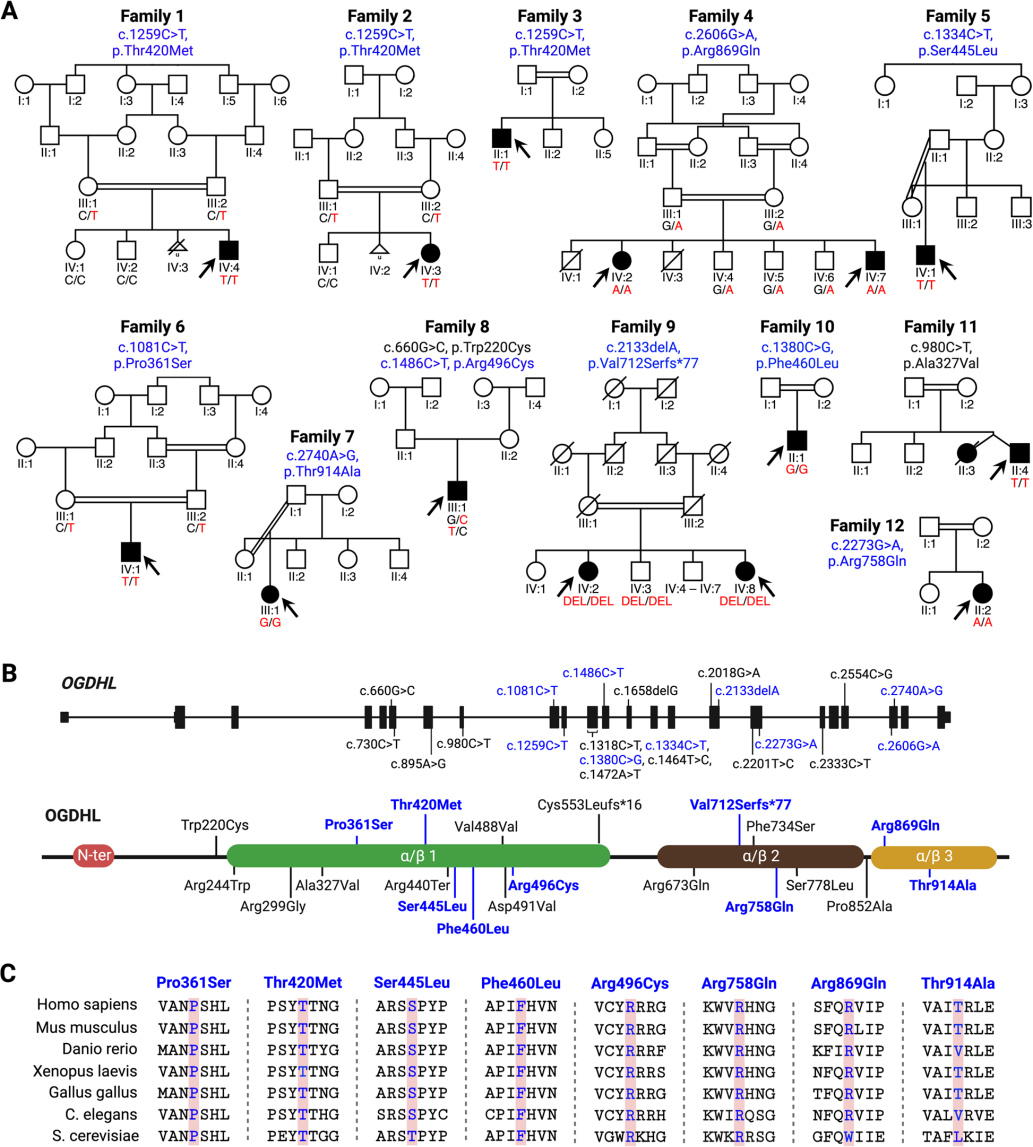
Identification of *OGDHL* variants in 12 families and mapping of known variants. **A** Pedigrees and segregation data for the 12 families included in this study. Affected and unaffected individuals are indicated by filled and open squares (males) and circles (females), respectively, and a triangle represents a pregnancy. Affected individuals are indicated by black arrows. Double lines indicate consanguinity. Genetic diagnoses were made in 14 individuals. **B** The position of changed coding sequence in genomic DNA and the drawing of resulting variants. Seven previously uncharacterized variants described in this study are marked in bold blue. **C** Seven out of eight missense variants from our studies are highly conserved across species

Using a genotype-first approach, 14 individuals (8 males, 6 females, ranging from 4 months to 43 years at last examination) from 12 independent families of African, European, Middle Eastern, and North African descent were identified based on the presence of biallelic *OGDHL* variants [13] (Fig. 1A). Proband-only or trio-exome sequencing revealed 10 missense and 1 frameshift variant in *OGDHL* (Additional file 3: Table S2). All individuals were homozygous except for one with compound heterozygous variants. Variants affected moderate to highly conserved (to *S. cervisiae*) amino acid residues (Fig. 1B (blue) and Fig. 1C). Additionally, review of candidate variants in other genes were classified in parallel (Table 1 and Additional file 4: Table S3). *OGDHL* variant classifications were performed before and after functional analysis (Table 1 and Additional file 3: Table S2); however, those in this section correspond to classifications that were done before zebrafish functional analysis.

**Table 1:**
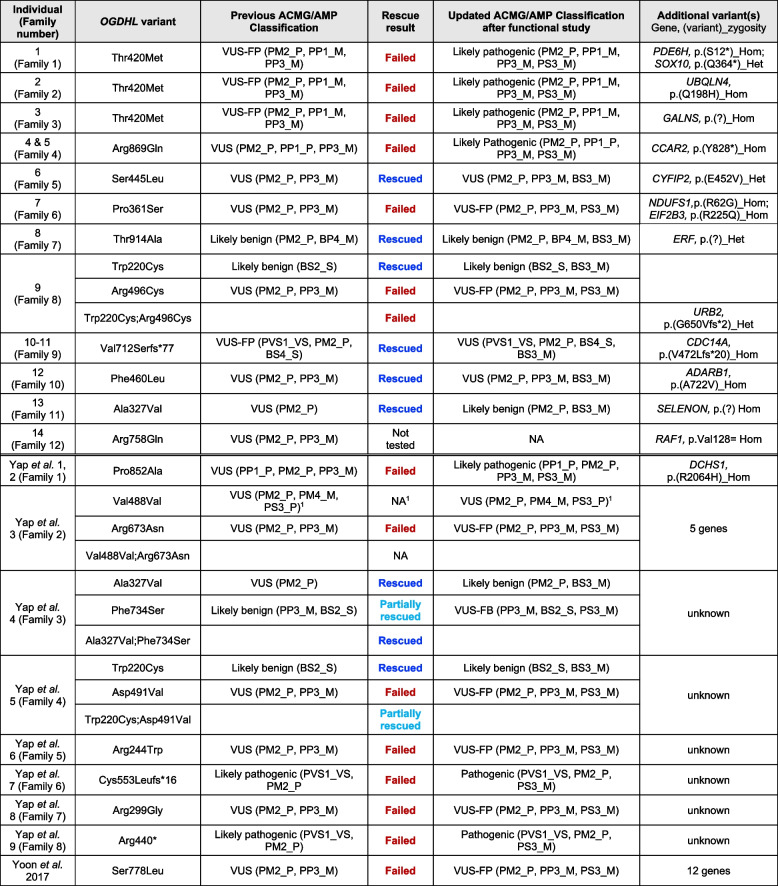
Summary of rescue results and an integration of previous and updated variant classification

The rescue results were concluded from Fig. 7B–C. Both eye and head size were significantly restored compared to the *ogdhl* F_0_ mutant embryos was classified as rescued (blue text). Only eye or head size was significantly restored was classified as partially rescued (light blue text). Neither eye nor head size was significantly restored was considered as failed rescue (red text)

*AD* Autosomal dominant, *AR* Autosomal recessive, *BP* Benign supporting, *BS* Benign strong, *Het* Heterozygous, *Hom* Homozygous, *M* Moderate, *P* Supporting, *PM* Pathogenic moderate, *PS* Pathogenic strong, *PVS* Pathogenic very strong, *S* Strong, *VS* Very strong. Pathogenic ≥ 10 points, likely pathogenic between 6 and 9 points, VUS-favor pathogenic = 5 points, VUS = 1-4 points, VUS-favor benign = 0 points, likely benign = -6 to -1 points, benign ≤ -7 points

^1^This variant was characterized as causing a leaky in-frame skipping of exon 11 that was also detected in controls to a lesser extent. Recognizing that the variant effect at hand involves aberrant splicing, we omitted the incorporation of rescue data in classification following functional testing, as our assay cannot evaluate splice effects

A homozygous c.1259C > T, p.(Thr420Met) variant was identified in individuals 1-3, of Iranian, Egyptian, and Sudanese genetic ancestries, respectively. Confirmatory segregation analysis was possible in two families, leading to an overall classification of VUS-FP (PM2_P, PP1_M, PP3_M, 5 points). Homozygosity mapping supported the identification and segregation of the c.2606G > A, p.(Arg869Gln) variant in individuals 4 and 5 that was classified as VUS (PM2_P, PP1_P, PP3_M, 4 points). Individuals 6 and 7 were homozygous for the c.1334C > T, p.(Ser445Leu), and c.1081C > T, p.(Pro361Ser) variants, respectively, each classified as VUS (PM2_P, PP3_M, 3 points). The homozygous c.2740A > G, p.(Thr914Ala) variant in individual 8 was classified as likely benign (PM2_P, BP4_M, -1 point). Individual 9 presented compound heterozygous variants, one of which was previously described c.660G > C, p.(Trp220Cys) [6] that was classified as likely benign (BS2_S, -4 points), while the c.1486C > T, p.(Arg496Cys) variant was classified as VUS (PM2_P, PP3_M, 3 points). The homozygous c.2133delA, p.(Val712Serfs*77) variant in individuals 10-11 was classified as VUS-FP and included criteria for one discordant segregation in a healthy sibling (PVS1_VS, PM2_P, BS4_S, 5 points). The homozygous c.1380C > G, p.(Phe460Leu) variant was identified in individual 12 and classified as VUS (PM2_P, PP3_M, 3 points). Individual 13 was homozygous for a previously published heterozygous c.980C > T, p.(Ala327Val) variant that was classified as VUS (PM2_P, 2 points) [6]. The homozygous c.2273G > A, p.(Arg758Gln) was identified in individual 14 and classified as VUS (PM2_P, PP3_M, 3 points).

### Clinical findings in the patient cohort with biallelic *OGDHL* variants

Detailed clinical information from all individuals is included in the Additional file 2: Supplemental Case Reports and summarized in Additional file 5: Table S4. A history of consanguinity was frequently observed (13/14). A family history was only reported in families 4, 8, 10, and 11. Similar to clinical features of previously reported patients [6], these patients presented a range of highly heterogeneous phenotypes with variable severity including neurodevelopmental disorders, neurodegeneration, infantile-onset epileptic encephalopathy, skeletal dysplasia, childhood-onset epilepsy, multiple congenital anomalies, dysmorphism, non-syndromic hearing loss, neuromuscular disorders, and congenital heart defects.

**Fig. 2:**
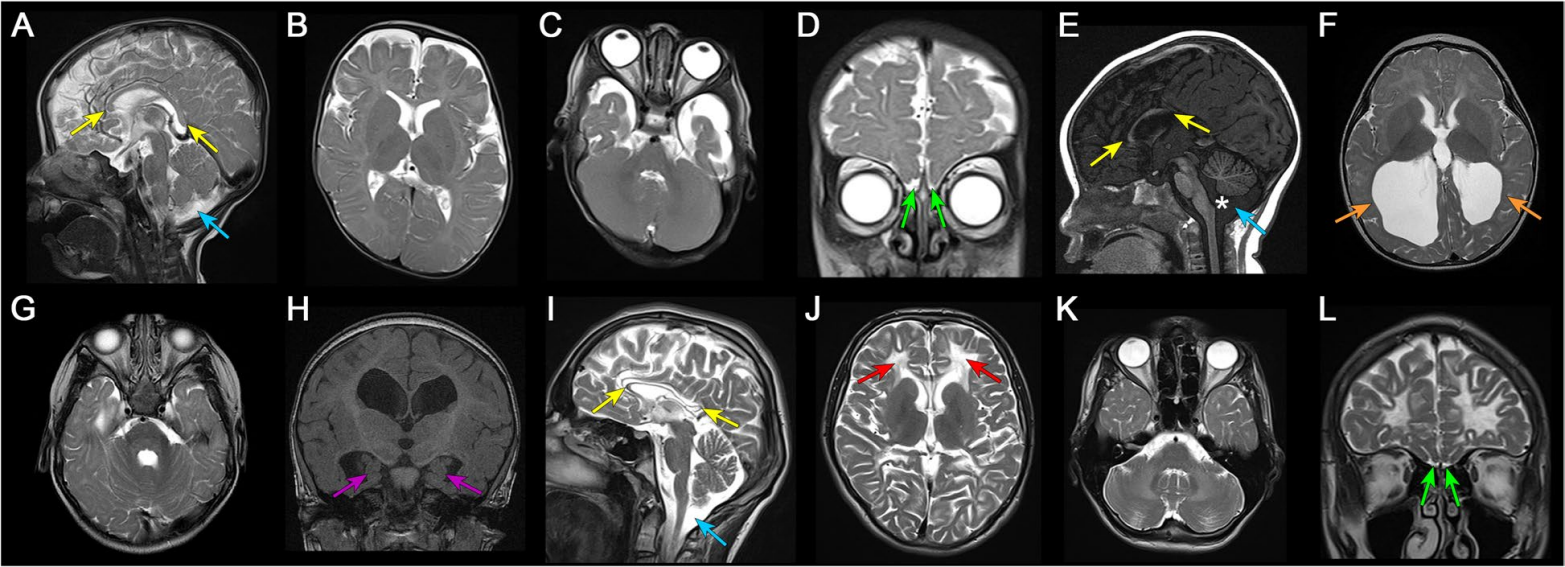
Neuroimaging findings of individuals with *OGDHL* pathogenic variants. Brain MRI findings of individual 1 (**A**-**D**), 6 (**E**–**H**), and 7 (**I**-**L**). Sagittal T2 (**A** and **I**) and T1 (**E**) weighted images showed markedly hypoplastic corpus callosum in individual 1 (**A**) and 7 (**I**) and dysplastic corpus callosum in individual 6 (**E**) with hypoplastic rostrum, genu, and anterior body and absent posterior body and splenium (yellow arrows). Mega cisterna magna was present in all affected individuals (**A**, **E**, and **I**, blue arrows), and individual 6 also had inferior vermian hypoplasia and widening of the foramen of Magendie (**E**, asterisk). Axial T2-weighted images (**B**, **C**, **F**, **G**, **J**, and **K**) revealed varying degrees of diffuse white matter volume loss, most severe in individual 6 (**F** and **G**) and 7 (**J** and **K**). Individual 7 also had scattered areas of leukomalacia (**J**, red arrows) and prominent involvement of brainstem and cerebellar white matter. Individual 6 had ventriculomegaly and colpocephaly (**F**, orange arrows). Coronal T2 (**D** and **L**) and T1 (**H**) weighted images showed hypoplastic olfactory bulbs in individual 1 (**D**) and 7 (**L**) (green arrows) and hypoplastic hippocampi in individual 6 (**H**, purple arrows)

However, the most frequently reported clinical features among these families were hypotonia (9/14), short stature and variable dysmorphic facial features (each 8/14), failure to thrive (7/14), as well as developmental delay/intellectual disability (9/14). Seizures (4/14), hearing loss (4/14), and microcephaly (3/14) were also observed in subset of the patients. Eight individuals had brain magnetic resonance imaging (MRI) studies; five were reviewed by a board certified neuroradiologist and all had abnormal findings, while three had reportedly normal studies (not available for independent review). Shared features included hypoplastic/dysplastic corpus callosum and anterior commissure, varying degrees of white matter volume loss ranging from mild to severe, and mild prominence of the ventricular system. Anteriorly rotated thalami and hypoplastic olfactory bulbs were each present in two cases (Fig. 2 and Additional file 5: Table S4).

As each individual had at least one additional variant identified (Additional file 4: Table S3), we dissected the possible contribution of these variants to a blended phenotype. Individual 1 had a heterozygous pathogenic *SOX10* (NM_006941.4:c.1090C > T, p.Gln364Ter) variant, likely accounting for hearing impairment, ocular albinism, and Hirschsprung disease as part of PCWH syndrome (peripheral demyelinating neuropathy, central dysmyelination, Waardenburg syndrome, and Hirschprung disease) (OMIM #609136) [27]. Individual 3 was suspected to have mucopolysaccharidosis. A homozygous VUS was identified in *GALNS* (NM_001323544.2:c.1158-3C > G, p.?) that causes mucopolysaccharidosis type 4A (OMIM #253000) potentially explaining short stature and abnormal facial and skeletal abnormalities. Individual 6 presented with a heterozygous VUS in *CYFIP2* (NM_001037333.3:c.1355A > T, p.(Glu452Val)) that was of unknown inheritance. Variants in this gene cause autosomal dominant developmental and epileptic encephalopathy 65 (OMIM #618008), developmental delay, and profound intellectual disability [28] and may account for seizures, spasticity, and the presence of brain abnormalities. Individual 7 had two variants: a homozygous *NDUFS1* VUS (NM_005006.7:c.184C > G, p.(Arg62Gly)) and a homozygous pathogenic *EIF2B3* variant (NM_020365.5:c.674G > A, p.(Arg225Gln)) [29]. Both genes may contribute to the presence of brain abnormalities on imaging studies and seizures. Biallelic variants in *NDUFS1* are associated with mitochondrial complex I deficiency, nuclear type 5 (OMIM #618226). Variants in *EIF2B3* cause a recessively inherited leukoencephalopathy with vanishing white matter (OMIM #620313). Individuals 10 and 11 had a pathogenic homozygous *CDC14A* variant (NM_033312.2:c.1421 + 2 T > C, p.Val472Leufs*20) that explains isolated hearing impairment (OMIM #608653) [30]. Individual 12 had a homozygous likely pathogenic variant in *ADARB1* (NM_015833.4:c.2165C > T, p.Ala722Val) that was associated with non-specific neuroimaging findings, seizures, and developmental delay [31]. Individual 13 had a homozygous pathogenic variant in *SELENON* (NM_020451.3:c.-11_81del, p.(Met1fs)) that explains myopathy (OMIM #606210) [32, 33]. All other identified variants impacted genes without an established link to hereditary diseases or genes with unknown or poorly characterized function (Additional file 4: Table S3). Given the presence of complicating additional variants in most patients, we turned to a genetic model to investigate brain pathology in a vertebrate system with highly conserved *OGDH* and *OGDHL* genes in order to determine whether and which of the clinical findings are likely caused by *OGDHL* biallelic variants.

### OGDHL homologs are highly conserved in sequence and expression between human and zebrafish

Zebrafish *ogdhl* (ENSDARG00000079249) encodes a 1008 amino acid protein with four conserved domains including 2-oxoglutarate dehydrogenase N-terminal, α/β1 domain (Thiamine diphosphate (ThDP) binding), α/β2 domain (Transketolase & pyrimidine binding), and α/β3 domain, like human OGDHL*.* The sequence alignment of zebrafish Ogdhl and human OGDHL with orthologs in other species shows high conservation (Additional file 2: Fig. S2A). Zebrafish and human OGDHL proteins are 78% identical and 87% similar (Additional file 2: Fig. S2B), and the two major functional domains, α/β1 and α/β2, share over 90% identity (Additional file 2: Fig. S2C). We also compared human OGDH and DHTKD1 with their orthologs in zebrafish including two Ogdh paralogs (Ogdha and Ogdhb) and Dhtkd1. Both phylogenetic analysis and protein sequence alignments show that zebrafish Ogdha and Ogdhb are highly homologous to human OGDH (> 80% identity and > 90% similarity; Additional file 2: Fig. S2D, E). Moreover, both human and zebrafish OGDHL/Ogdhl show > 70% identity and > 80% similarity with human OGDH (Additional file 2: Fig. S2E), suggesting potential functional redundancy. In contrast, human DHTKD1 and zebrafish Dhtkd1 proteins are less similar in sequence to human OGDH (Additional file 2: Fig. S2D, E). To examine the gene expression profile of *ogdh* family members during development, we used RT-qPCR and compared mRNA expression with existing expression data from different embryonic developmental stages in zebrafish [34]. Our analysis revealed that *ogdha*, *ogdhb*, and *dhtkd1* are expressed maternally, while *ogdhl* mRNA is only detected from 48 hours post-fertilization (hpf) stage onwards and gradually increases during development (Additional file 2: Fig. S3A). Expression analysis of various tissues further demonstrated an enrichment of *ogdhl* mRNA in zebrafish neuronal tissues such as the brain, eye, and spinal cord (Additional file 2: Fig. S3B), in agreement with previous reports suggesting a notable expression of both mRNA and protein in the rat brain and spinal cord [35], as well as across all regions of the human brain [36]. Moreover, a parallel observation was made through single-cell transcriptome analysis, which revealed similar enrichment in the human and porcine ocular compartments (Additional file 2: Fig. S3C), as well as the mouse brain (Additional file 2: Fig. S3D). Therefore, these results support the conclusion that zebrafish is an appropriate animal model to investigate *OGDHL* variant pathogenicity.

### Zebrafish* ogdhl *F_0_ knockout recapitulates a subset of clinical features

**Fig. 3:**
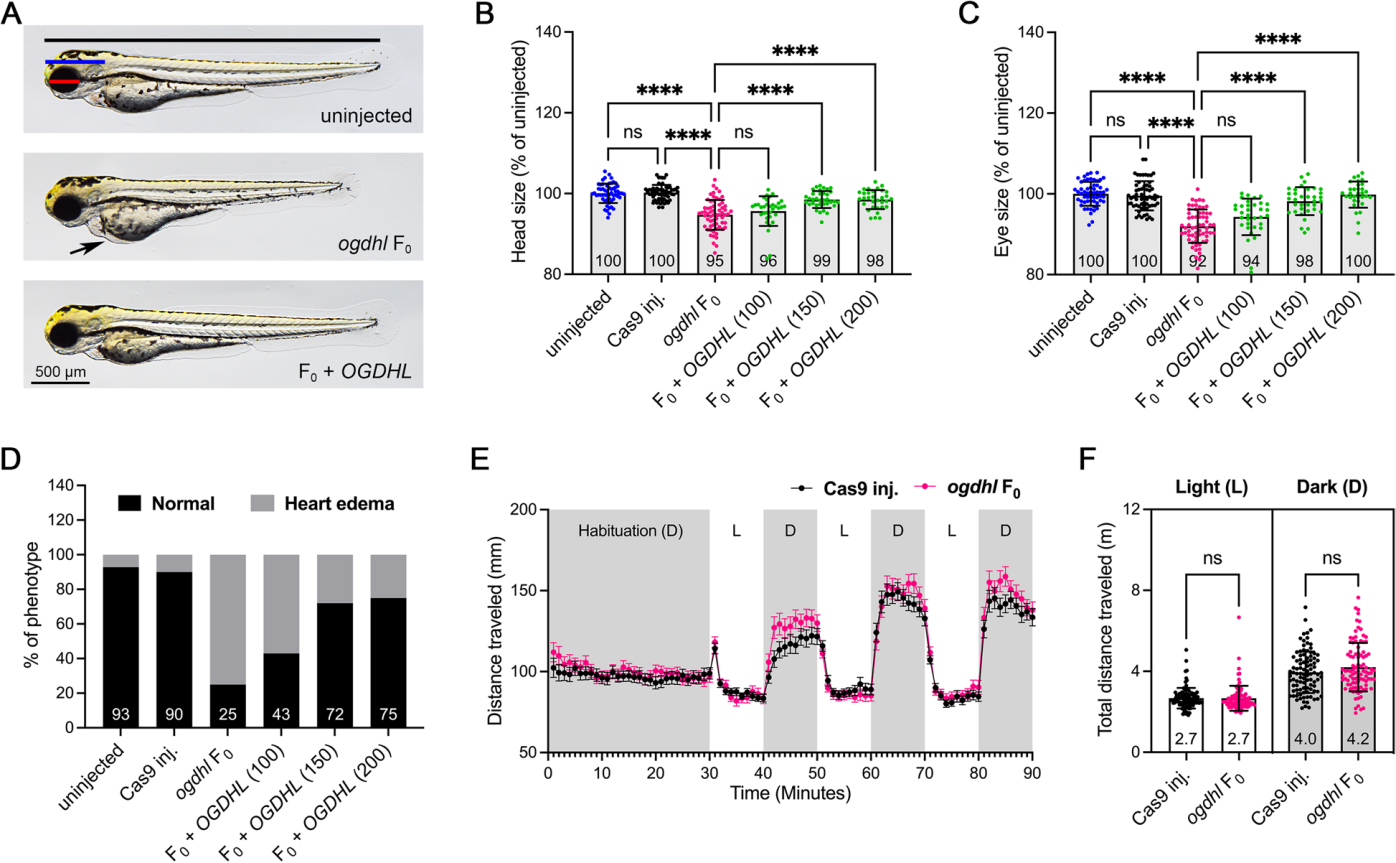
The morphological phenotypes of zebrafish *ogdhl* F_0_ knockouts. **A** Representative embryo images of uninjected, *ogdhl* F_0_ and *ogdhl* F_0_ co-injected with 200 picogram (pg) human *OGDHL* mRNA (F_0_ + *OGDHL*) at 3 dpf. Arrow denotes heart edema. **B**,**C** Size measurements for head (blue line) and eye (red line) as indicated from genotypes in **A** were calculated as a percentage difference compared to the mean value of the uninjected embryos. **D** The presence of heart edema (black arrow in **A**) was calculated as a percentage of total embryos of each group positive for edema. **E** Locomotor activities of zebrafish larvae in light and dark conditions at 5 dpf, *n* = 96 larvae for each group. The larvae were habituated in the dark for 30 min, followed by three cycles of 10-min periods of light and dark. Error bars represent the mean ± SEM. Dark period (**D**), light period (**L**). **F** Average cumulative distance traveled by each larva during three cycles of either light or dark periods. Error bars = mean ± SD. For **B**, **C**, and **F**, each dot represents one embryo, and the mean value of each group is indicated at the bottom of the respective bar in the figure. For **B–D**, the number of embryos for uninjected = 57, Cas9 protein-injected (Cas9 inj.) = 60, *ogdhl* F_0_ = 63, F_0_ + *OGDHL* (100 pg) = 34, F_0_ + *OGDHL* (150 pg) = 35 and F_0_ + *OGDHL* (200 pg) = 32. Error bars = mean ± SD. Statistical significance was calculated by Brown–Forsthye and Welch’s ANOVA with Dunnett’s T3 multiple comparisons test: not significant (ns) *p* ≥ 0.05, *p < 0.05, ***p < 0.001, and ****p < 0.0001

To investigate the in vivo function of Ogdhl, we targeted *ogdhl* using CRISPR/Cas9 genome editing. This approach generated a 4-bp deletion allele that was selected for phenotypic assessment of the F2 generation in stable genetic knockouts. Stable *ogdhl* mutant animals only showed subtle morphological phenotypes (Additional file 2: Fig. S4A), prompting us to investigate whether genetic compensation is occurring in these mutants. This is particularly relevant, as it has been shown that PTC (premature termination codon)-bearing indels can activate genetic compensation responses leading to an increase in the expression of related genes within the same biological pathway or functional group [37–39]. This compensatory mechanism helps to rescue the developmental defects caused by deleterious variants by either partially or entirely compensating for the loss of the targeted gene's function. Due to this possibility, we investigated a comprehensive exploration of potential genetic compensation responses in our stable *ogdhl* mutant models (Additional file 2: Supplementary Results and Fig. S4-5). This uncovered substantial upregulation of *OGDHL* paralogs identified via RT-qPCR in stable genetic mutants (Additional file 2: Fig. S4B, C). Therefore, we utilized an *ogdhl* F_0_ knockout model, which has been demonstrated to not robustly activate genetic compensation, resulting in phenotypes with more relevance to those observed in affected individuals [39, 40]. Indeed, unlike the strong upregulation (64-fold) of *dhtkd1* expression in *ogdhl*-/- mutants (Additional file 2: Fig. S4B, C), we observed only a slight increase (threefold) in *dhtkd1* and a strong reduction in *ogdhl* mRNA levels in *ogdhl* F_0_ knockouts compared to uninjected controls (Additional file 2: Fig. S6A). Furthermore, we observed that *ogdhl* F_0_ animals exhibited smaller head, eye, and body sizes (Fig. 3A-C and Additional file 2: Fig. S6B), as well as heart edema (Fig. 3D) when compared to either uninjected or Cas9-injected animals at 3 dpf. The phenotypes observed in the *ogdhl* F_0_ knockouts are in alignment with those of *ogdhl*-/- animals, albeit more severe. To confirm if the observed phenotypes are caused by the loss of Ogdhl, we employed a rescue approach using capped mRNA encoding human OGDHL protein at three different concentrations (100, 150, and 200 pg). Injection of human *OGDHL* led to a dose-dependent rescue of phenotypes such as the eye, head, and body size, and heart edema (Fig. 3B-D). This result provides robust evidence that the observed phenotypes are caused by the absence of Ogdhl and confirms that *ogdhl* has conserved functions in zebrafish and human.

The neurodevelopmental disorders associated with *OGDHL* variants are highly clinically heterogeneous, necessitating careful dissection of the phenotypes directly associated with *OGDHL.* A previous study reported that approximately half of affected individuals displayed seizures; in our study, of the 14 affected individuals, 4 reported seizures. Among these 4 individuals, 3 presented additional variants in genes known to be associated with seizures, raising the question of whether seizure is a phenotype specifically related to *OGDHL* loss-of-function. To address this, we conducted locomotion behavior tests on *ogdhl* F_0_ knockouts using 10-min intervals of light–dark cycles [41]. There was no statistically significant difference in the total distance traveled between *ogdhl* F_0_ and Cas9-injected animals (Fig. 3E, F), although we observed a slight increase in *ogdhl* F_0_ knockouts. Additionally, we assessed the visual and hearing abilities of *ogdhl* F_0_ knockouts and found neither affected (Additional file 2: Fig. S6C, D). Based on these results, we conclude that loss of OGDHL may not directly result in seizure manifestation, visual impairment, or hearing deficiencies. However, it is important to note that we cannot completely exclude the possibility that these animals may have a heightened susceptibility to developing such conditions.

### Complex functional redundancy of OGDH, OGDHL, and DHTKD1 isoenzymes

**Fig. 4:**
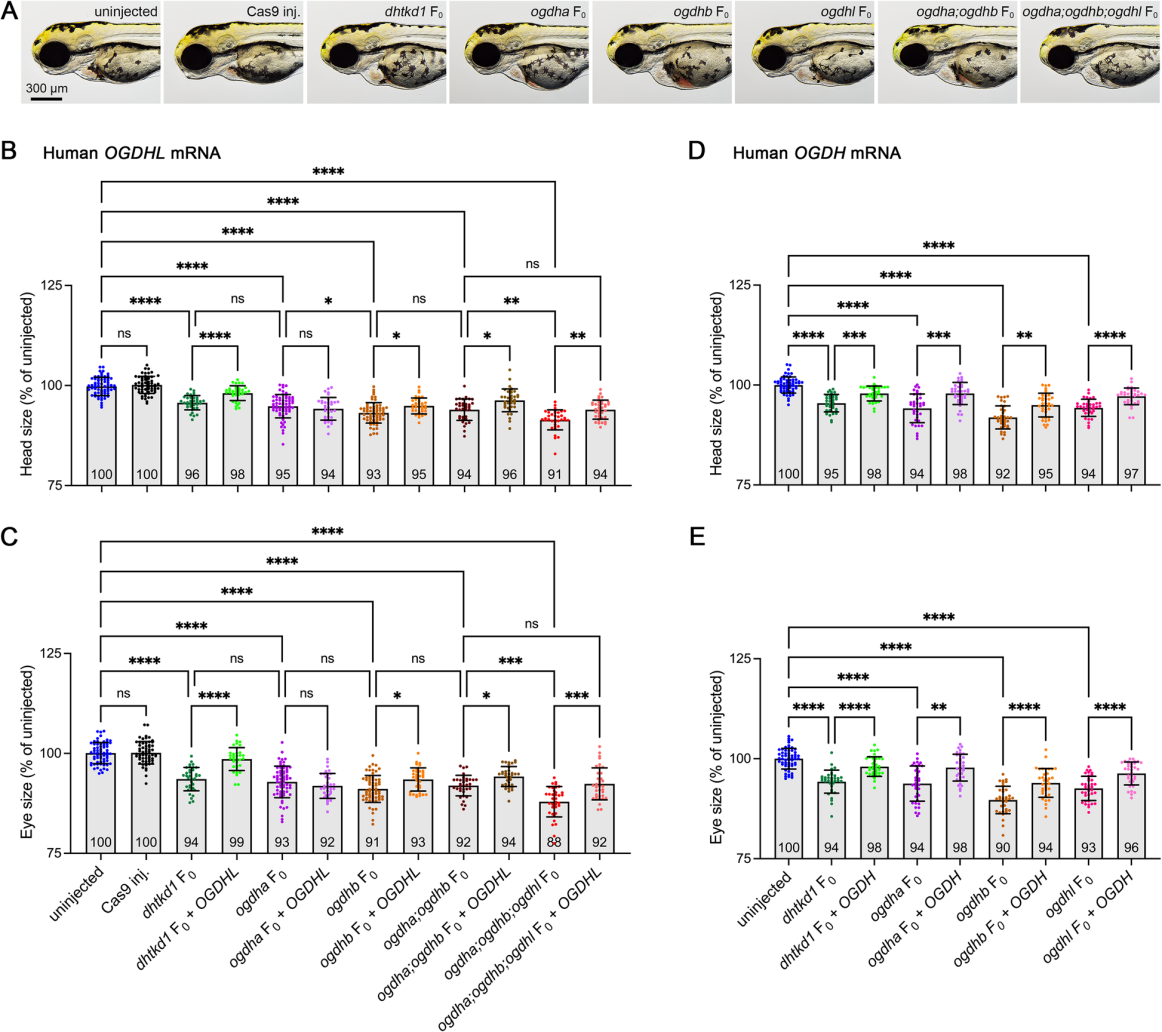
Comparing mRNA rescue levels of OGDHL and OGDH in Dhtkd1 and Ogdh paralog-deficient zebrafish. **A** Representative image of uninjected, Cas9-injected, single knockout (*dhtkd1*, *ogdha*, and *ogdhb* F_0_), double knockout (*ogdha;ogdhb* F_0_), and triple knockout (*ogdha;ogdhb;ogdhl* F_0_) embryos at 3 dpf. **B**, **C** Size measurements for head and eye with or without human OGDHL co-injection. The number of embryos for uninjected = 66, Cas9-injected = 60, *dhtkd1* F_0_ = 36, *dhtkd1* F_0_ + *OGDHL* = 36, *ogdha* F_0_ = 66, *ogdha* F_0_ + *OGDHL* = 36, *ogdhb* F_0_ = 66, *ogdhb* F_0_ + *OGDHL* = 36, *ogdha;ogdhb* F_0_ = 36, *ogdha;ogdhb* F_0_ + *OGDHL* = 36, *ogdha;ogdhb;ogdhl* F_0_ = 36 and *ogdha;ogdhb;ogdhl* F_0_ + *OGDHL* = 36. **D**, **E** The size measurements for head and eye with or without human OGDH co-injection. The number of embryos for uninjected = 60, *dhtkd1* F_0_ = 36, *dhtkd1* F_0_ + *OGDH* = 36, *ogdha* F_0_ = 36, *ogdha* F_0_ + *OGDH* = 36, *ogdhb* F_0_ = 36, *ogdhb* F_0_ + *OGDH* = 36, *ogdhl* F_0_ = 36, and *ogdhl* F_0_ + *OGDH* = 36. All values were calculated as a percentage difference compared to the mean value of the uninjected embryos. Each dot represents one embryo, and the mean value of each group is indicated at the bottom of the respective bar in the figure. Error bars = mean ± SD. Statistical significance was calculated by Brown–Forsthye and Welch’s ANOVA with Dunnett’s T3 multiple comparisons test: not significant (ns) *p* ≥ 0.05, *p < 0.05, **p < 0.01, ***p < 0.001, and ****p < 0.0001

OGDHL and OGDH have similar structures and substrate profiles and have been suggested to have similar enzymatic functions [7, 8]. Furthermore, a previous in vitro study has shown that OGDH displays partial activity compensating for degradation of the DHTKD1 substrate, 2-oxoadipic acid [9]. Variants in *OGDH*, *OGDHL*, and *DHTKD1* isoenzymes have been associated with various clinical phenotypes, including microcephaly [6, 10, 42]. While there is evidence for similar or partially overlapping catalytic functions of all three isoenzymes, it is unknown whether each can compensate for the morphological phenotypes resulting from isoenzyme gene loss in whole organisms. We generated F_0_ knockouts for *dhtkd1*, *ogdha*, and *ogdhb* using CRISPR/Cas9 and co-injected with human *OGDHL* mRNA (Fig. 4A-C). Our results showed that all F_0_ knockouts exhibited reduced head and eye size (Fig. 4A, B). Interestingly, *OGDHL* mRNA only rescued the phenotypes resulting from loss of Dhtkd1 or Ogdhb, showing the most efficacy in rescuing Dhtkd1 loss (Fig. 4B, C). RT-qPCR analysis confirmed human *OGDHL* expression in injected embryos at 3 dpf (Additional file 2: Figure S7A). Additionally, we generated *ogdha;ogdhb* double knockouts and *ogdha;ogdhb;ogdhl* triple knockouts. Interestingly, the phenotypes of *ogdha;ogdhb* double knockouts were no more severe than *ogdhb* single knockouts; however, *ogdha;ogdhb;ogdhl* triple knockouts had the most severe effects of all tested genotypes. We infer that Ogdha and Ogdhb may have redundant roles that are distinct from that of Ogdhl in neurodevelopment. Moreover, co-injection of *OGDHL* in *ogdha;ogdhb;ogdhl* triple knockouts only rescued phenotypes back to *ogdha;ogdhb* double knockout levels, further supporting the distinct roles of OGDH vs. OGDHL. Reciprocally, we investigated the genetic redundancy of OGDH in vivo using human *OGDH* mRNA co-injection and found that it effectively rescued the phenotypes resulting from the loss of each isoenzyme gene (Fig. 4D, E and Additional file 2: Fig. S7B). Thus, OGDH is uniquely necessary and sufficient for proper zebrafish neurodevelopment.

Taken together, our data provide evidence for a complex compensatory relationship between 2-oxoglutarate (OGDH(L)) and 2-oxoadipate (DHTKD1) dehydrogenases, underscoring their interconnected roles in developmental processes. Importantly, our findings emphasize the critical and non-redundant role of OGDH, further establishing its indispensability in regulating development.

### Loss of Ogdhl causes neuronal cell apoptosis and motor neuron defects

**Fig. 5:**
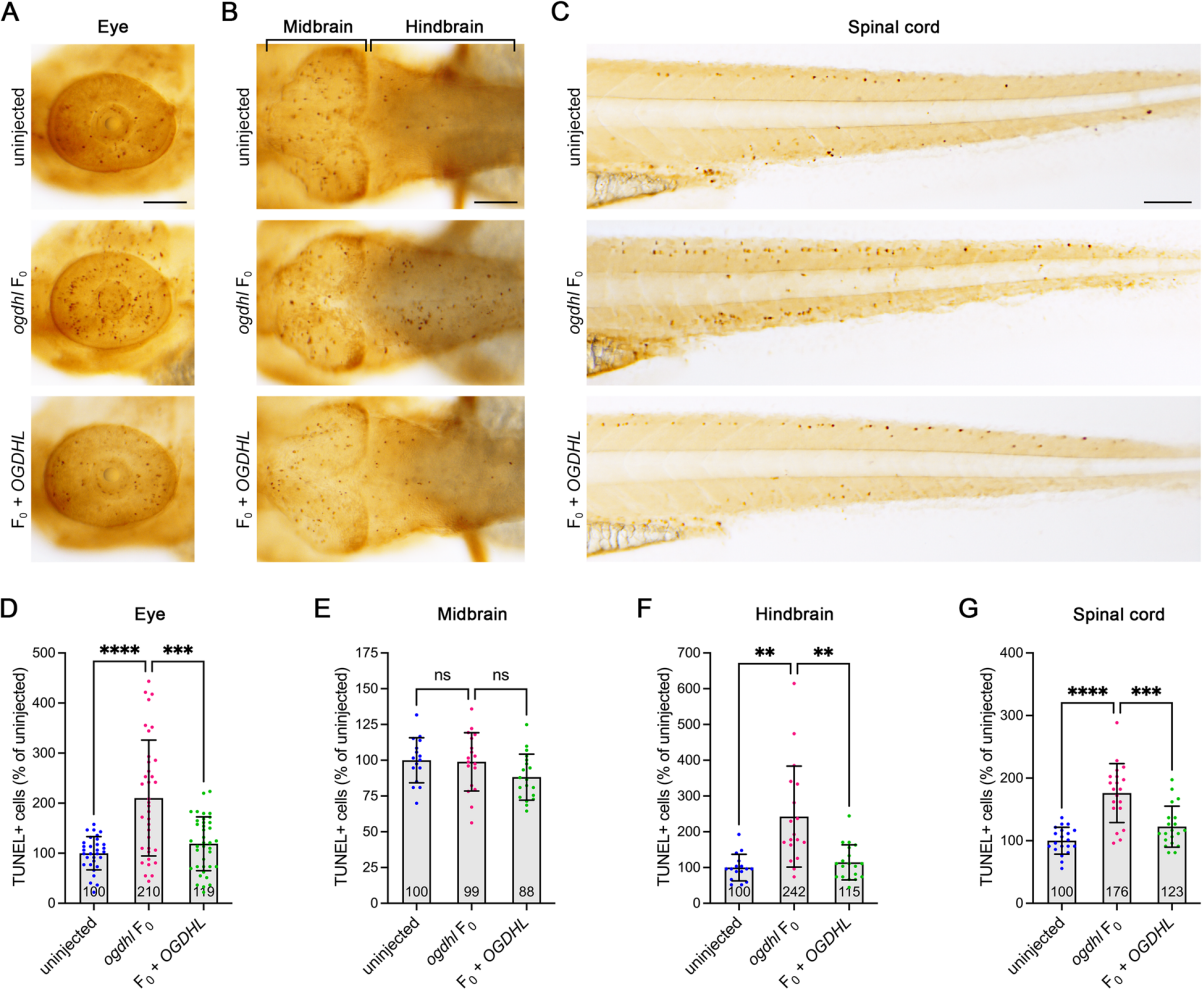
Cell apoptosis was activated by *ogdhl* loss-of-function. **A–C** Representative image of uninjected, *ogdhl* F_0_ and *ogdhl* F_0_ co-injected with human *OGDHL* embryos at 3 dpf after TUNEL staining. Scale bars = 100 μm. **A** Lateral view of eyes. Anterior to the left and dorsal to the top. **B** Dorsal view of brains. Anterior to the left. **C** Lateral view of trunks. Anterior to the left and dorsal to the top. **D–G** Quantification of the number of TUNEL-positive cells in the eye, midbrain, hindbrain, and spinal cords. The mean value of each group is indicated at the bottom of the respective bar in the figure. TUNEL-positive cells were calculated as a percentage difference compared to the mean value of uninjected embryos. In **D**, each dot represents one eye. uninjected = 31, *ogdhl* F_0_ = 36 and F_0_ + *OGDHL* = 38 eyes. In **E–G**, each dot represents one animal. uninjected = 16, *ogdhl* F_0_ = 18 and F_0_ + *OGDHL* = 19 animals. Error bars = mean ± SD. Statistical significance was calculated by Brown–Forsthye and Welch’s ANOVA with Dunnett’s T3 multiple comparisons test: not significant (ns) *p* ≥ 0.05, **p < 0.01, ***p < 0.001, and ****p < 0.0001

In light of the wide range of neurological symptoms observed in individuals affected by *OGDHL* variants, such as intellectual disability and hypotonia, and considering the high expression of *OGDHL* in the brain, we focused on examining neuronal tissues in more depth. Previous research demonstrated the crucial role of OGDH in neuronal cell death, which is associated with neurodegenerative diseases [43, 44]. Therefore, we conducted a terminal deoxynucleotidyl transferase (TdT) dUTP Nick-End Labeling (TUNEL) assay to investigate whether the observed morphological defects in animals lacking Ogdhl are caused by abnormal cell apoptosis. We observed a notable increase in TUNEL-positive cells in the eye, hindbrain, and spinal cord in F_0_ knockouts compared to control animals (Fig. 5A–G). However, no significant change in TUNEL-positive cells was observed in the midbrain region, suggesting that specific cell types are sensitive to Ogdhl loss. Importantly, we observed a significant reduction in TUNEL-positive cells in animals co-injected with human *OGDHL* mRNA. Quantification of the expression of apoptotic-related genes using RT-qPCR showed significant upregulation of *casp8*, *casp9*, and *mdm2* in *ogdhl* F_0_ animals, which was also alleviated upon co-injection with human *OGDHL* mRNA (Additional file 2: Fig. S8A). It has been shown that during neuronal cell apoptosis, the expression of not only active Caspase-8 and active Caspase-9 but also c-Fos (*fosab* in zebrafish) is elevated [45]. Therefore, we measured the levels of *fosab* in *ogdhl* F_0_ knockouts and found that *ogdhl* F_0_ displayed elevated *fosab* mRNA levels, which can be rescued by overexpressing *OGDHL* (Additional file 2: Fig. S8B). Taken together, these findings suggest that elevated neuronal cell death could be responsible for the neurological phenotypes observed in individuals with biallelic variants in *OGDHL*.

**Fig. 6:**
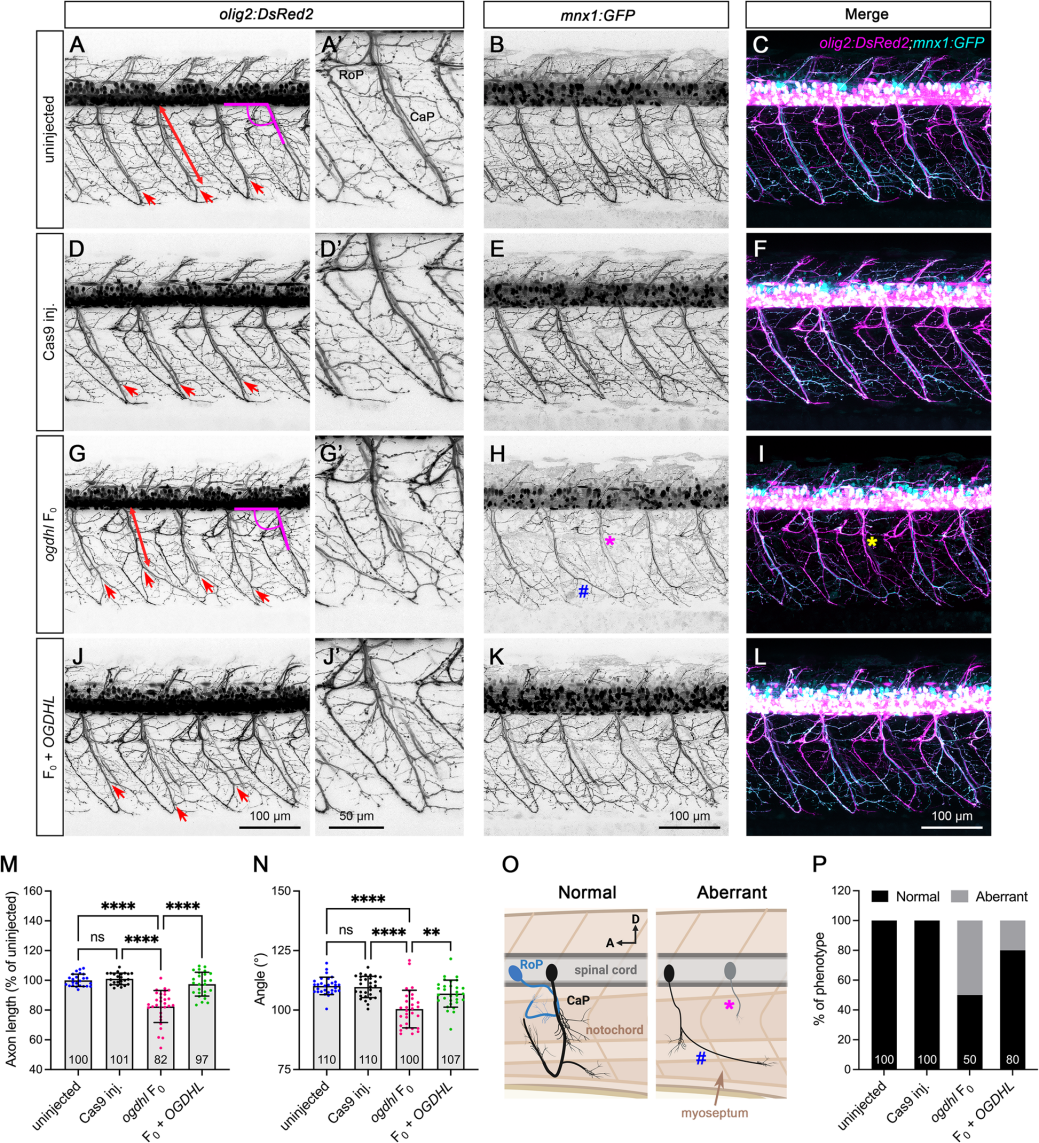
Zebrafish *ogdhl* F_0_ mutant displays motor neuron abnormalities. **A–L** Representative image of the trunk region of uninjected, Cas9-injected, *ogdhl* F_0_ and F_0_ + *OGDHL* in *Tg(olig2:DsRed2;mnx1:GFP);Albino* embryos at 3 dpf. **M** The measurement of axon length was started from spinal cord to the end of latest posterior branch (indicated by red arrow) of caudal primary motor neurons (CaP) (indicated by red line). Axon lengths were calculated as a percentage difference compared to the mean value of uninjected embryos. Each dot represents one axon. Uninjected = 24, Cas9-injected = 24, *ogdhl* F_0_ = 30, and F_0_ + *OGDHL* = 26. **N** The measurements of axon angle (indicated by magenta lines, start from spinal cord). Each dot represents one axon. *n* = 30 axons for each group. Error bars = mean ± SD. The mean value of each group was indicated at the bottom of the respective bar in the figure. Statistical significance in **M** and **N** was calculated by Brown–Forsthye and Welch’s ANOVA with Dunnett’s T3 multiple comparisons test: not significant (ns) *p* ≥ 0.05, **p < 0.01, and ****p < 0.0001. **O** Cartoon figures indicate aberrant motor axons observed in *ogdhl* F_0_ embryos such as cross somite segment (blue hash) or missing EGFP signal (magenta asterisk). **P** Quantification of aberrant motor axon as indicated in **O**. *n* = 30 axons for each group

To study the impact of OGDHL loss on motor neuron development, we generated *ogdhl* F_0_ in a transgenic zebrafish line, *Tg(olig2:DsRed2;mnx1:GFP)*, in which RFP is expressed in oligodendrocyte progenitor cells, primary motor neurons, and interneurons [46] and GFP is expressed in primary motor neurons [47] (Fig. 6). We focused on observation of the morphology of motor neurons, as abnormalities in their structure have been linked to symptoms such as hypotonia, spasticity, muscle weakness, and atrophy, which are also observed in affected individuals [48, 49]. Confocal imaging revealed that the caudal primary motor neurons (CaP) in *ogdhl* F_0_ were shorter (Fig. 6G, M) and thinner (Fig. 6H) compared to either uninjected or Cas9-injected control animals at 3 dpf, this phenotype was rescued upon overexpressing human *OGDHL* mRNA (Fig. 6J–L). Moreover, we also observed a decreased angle between CaP and the spinal cord in F_0_ animals (Fig. 6N), as well as aberrant CaP phenotypes (Fig. 6O, P) including missing *mnx1*-positive signal (Fig. 6H, I, asterisk) or pathfinding error (Fig. 6H, hashtag) [50]. These data indicate that loss of Ogdhl affects axon development of the primary motor neurons in zebrafish and might lead to movement disorders seen in *OGDHL* affected individuals.

### In vivo functional characterization of* OGDHL *variants

**Fig. 7:**
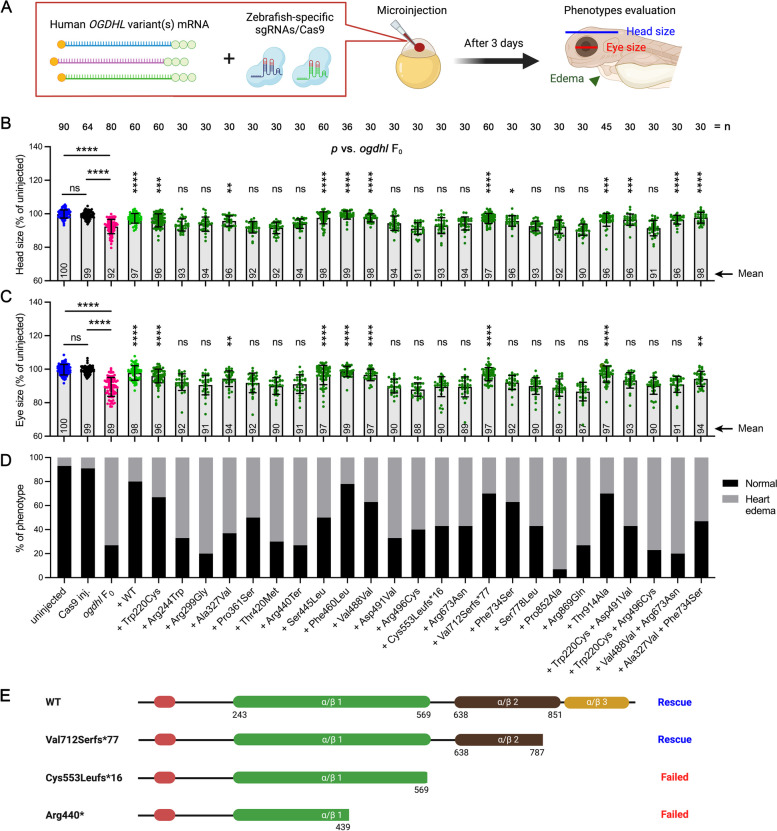
Functional characterization of human *OGDHL* variants in zebrafish model. **A** Experimental approach for characterizing functionality of human *OGDHL* variants. Human *OGDHL* variant mRNAs were mixed with synthetic zebrafish-specific *ogdhl* single-guide RNAs (sgRNAs) and Cas9 protein and microinjected into one-cell stage embryos, followed by phenotypic evaluation at 3 dpf. Blue line indicates head size measurement approach. Red line indicates eye size measurement. Green arrowhead indicates heart edema. **B**, **C** Head and eye size measurements were calculated as a percentage difference compared to the mean value of the uninjected embryos. Subsequently, the calculated measurements of uninjected, Cas9-injected, and *ogdhl* F_0_ embryos were compared. Additionally, the *ogdhl* F_0_ co-injected with either WT or mutated *OGDHL* mRNA were compared to the *ogdhl* F_0_ knockout embryos. Each dot represents one animal and the mean value of each group is indicated at the bottom of the respective bar in the figure. *n* = the number of embryos. Error bars = mean ± SD. Statistical significance was calculated by Brown–Forsthye and Welch’s ANOVA with Dunnett’s T3 multiple comparisons test: not significant (ns) *p* ≥ 0.05, *p < 0.05, **p < 0.01, ***p < 0.001, and ****p < 0.0001. Significances for all rescue experiments are in comparison to *ogdhl* F_0_. **D** The presence of the heart edema phenotype was calculated as a percentage of total embryos of each group. **E** The schematic of OGDHL truncating variants and the corresponding rescue experiment results

Molecular modeling suggested potential structural roles for the affected residues in our cohort (Additional file 2: Supplementary Results and Fig. S9). To experimentally determine the effect of these variants, they were functionally tested in the *ogdhl* F_0_ knockout zebrafish model. Thus far, 20 *OGDHL* variants have been identified in affected individuals [6]. This study describes 11 variants, including two previously described variants and one recurrent variant in three unrelated individuals. Only three variants are predicted to be null alleles (p.(Arg440Ter), p.(Cys553Leufs*16) and p.(Val712Serfs*77)). However, we hypothesized that missense variants could be hypomorphic, resulting in reduced protein function often comparable to the loss-of-function phenotypes of the knockout model and their ability to rescue knockout could be used as a proxy for protein functionality. We generated cDNAs with all published and most newly described variants. To functionally test the variants, the mRNA encoding human *OGDHL* cDNA carrying individual variants was microinjected into one-cell stage embryos together with sgRNAs targeting the *ogdhl* gene and Cas9 protein. The eye, head size, and pericardial edema phenotypes were measured, quantified, and compared with the *ogdhl* F_0_ mutants at 3 dpf (Fig. 7A). We also performed RT-qPCR to confirm the reduction of zebrafish *ogdhl* expression (Additional file 2: Fig. S10A) and faithful expression of human variants (Additional file 2: Fig. S10B) at similar levels in injected embryos across multiple variant rescue experiments at 3 dpf.

In our new cohort, six variants were initially classified as VUS [51] (Table 1 and Additional file 3: Table S2), among which two out of three failed to rescue (p.Pro361Ser and p.Arg496Cys) and were thus reclassified as VUS-FP (a change from 3 to 5 points), while the p.Arg869Gln variant was reclassified as likely pathogenic (4 to 6 points). Also failing to rescue was the p.Thr420Met variant that was reclassified from VUS-FP to likely pathogenic (5 to 7 points).

The phenotype rescue with the p.Ser445Leu and p.Phe460Leu (both 3 to 1 point), as well as p.Thr914Ala (-1 to -3 points), and p.Trp220Cys (-4 to -6 points) substitutions did not impact the original classifications of VUS and likely benign, respectively, while the p.Val712Serfs*77 frameshift variant was downgraded from VUS-FP to VUS (5 to 3 points). Interestingly, corroboration of this reclassification comes from the discordant segregation in family 9, unaffected individual IV:3, who has a homozygous variant (p.Val712Serfs*77) (Fig. 1A, Family 9). This suggests that variants in OGDHL with a partially truncated α/β2 domain at 3’ end retains functionality, unlike the fully truncated variants (p.Cys553Leufs*16 and p.Arg440*) (Fig. 7E). The p.Ala327Val was downgraded from VUS to likely benign (1 to -1 point). The substitutions p.Trp220Cys and p.Arg496Cys occurred in compound heterozygosity in individual 9. Only p.Trp220Cys partially rescued knockout phenotypes, indicating that the protein might be hypomorphic. To mimic the compound heterozygous condition, we combined p.Trp220Cys and p.Arg496Cys variant mRNAs and functionally tested in vivo. Rescue experiments showed the combined variants failed to rescue, suggesting the p.Arg496Cys variant is pathogenic and causal for loss-of-function phenotypes.

Eight previously published substitutions also failed to rescue knockout phenotypes (Fig. 7B–D and Table 1). These results allowed us to reclassify five variants from VUS to VUS-FP, including p.Arg673Asn, p.Asp491Val, p.Arg244Trp, p.Arg299Gly, and p.Ser778Leu (3 to 5 points), as well as one VUS to likely pathogenic (p.Pro852Ala, 4 to 6 points), and two likely pathogenic classifications to pathogenic for p.Cys553Leufs*16 and p.Arg440* (9 to 11 points). The p.Phe734Ser substitution showed partial rescue and reclassification from likely benign to VUS-FB (-2 to 0 points). Of note, a synonymous substitution p.(Val488Val) has shown leaky splicing of in-frame exon skipping and a ~ 35% reduction in *OGDHL* transcript compared to unaffected individuals [6]; however, this study used mRNA microinjection which does not recapitulate effects on transcription and mRNA processing that may affect gene expression in the patient. As our rescue data do not allow for the assessment of aberrant leaky splicing, we acknowledge this as a limitation and did not modify variant classification following rescue studies (VUS 4 points) (Table 1 and Additional file 3: Table S2).

Many of the described variants were found in compound heterozygosity in affected individuals. As previous studies did not test these variants together [6], we tested whether the combination of these variants would have a similar or different effects on phenotypes. The variants p.Trp220Cys (likely benign) and p.Asp491Val (VUS-FP) partially rescued the phenotypes when tested together. We therefore conclude that these genotypes most likely produce hypomorphic protein function and mild phenotypes.

ACMG/AMP guidelines provide a framework for variant classification and consider evidence categories that include population frequency data, variant type, and case-level data [51, 52]. These criteria allow refinement of classifications based on functional assay results and encourage conservative application of evidence weight, the rationale for application of moderate (PS3_M/BS3_M) instead of strong weight. In this study, we reclassify 15/20 of all *OGDHL* variants reported to date through functional rescue experiments (Table 1). The upgraded classifications included VUS to VUS-FP (*n* = 7), VUS-FP to likely pathogenic (*n* = 1), VUS to likely pathogenic (*n* = 2), likely benign to VUS-FB (*n* = 1), and likely pathogenic to pathogenic (*n* = 2). Two variants were downgraded (VUS-FP to VUS and VUS to likely benign), four showed no change (*n* = 2 likely benign, *n* = 2 VUS), and one synonymous variant causing a splice effect was unable to be assessed via our methodology and remained as VUS.

All but two homozygous variants in our cohort failed to rescue *ogdhl* loss-of-function phenotypes. The p.Ser445Leu variant was classified as VUS and the affected individual shows normal behavior, vision, and hearing. Similarly, the p.Thr914Ala variant significantly rescued the mutant phenotypes suggesting preserved function, concordant with its likely benign classification. However, in this case, the functional data is not correlated with clinical phenotypes in the affected individual. A complicating factor, however, is the use of alcohol and marijuana during the pregnancy of individual 8 (family 7, p.Thr914Ala homozygous), which can have severe health ramifications for a developing fetus such as facial abnormalities, growth retardation, heart and kidney malformations, as well as CNS abnormalities, potentially explaining some or all of their clinical presentation [53–55]. Additionally, individual 8 also has a pathogenic heterozygous splicing variant in *ERF* with similar aberrant splicing effect to a reported ClinVar case with craniosynostosis (accession: VCV001224302.2). *ERF* is associated with autosomal dominant craniosynostosis 4 (OMIM: 600775) and Chitayat syndrome (OMIM: 617180).

Three *OGDHL* variants partially rescued both eye and head phenotypes. The p.Trp220Cys variant remained likely benign following rescue experiments. Additionally, this variant has been found in a homozygous state in many healthy individuals. The p.Ala327Val variant was reclassified from VUS to likely benign, as it was able to rescue both eye and head size phenotypes in zebrafish. The p.Phe734Ser variant was upgraded from likely benign to VUS-FB, although neuronal knockout flies showed lethality. Partial rescue was observed in zebrafish, allowing application of the PS3_M code, but this was not enough evidence to change the overall classification.

We also used our model to test these variants in compound heterozygosity to reflect the genotypes of affected individuals. The p.Ala327Val/p.Phe734Ser, as initially described, showed stronger rescue than the p.Trp220Cys/p.Asp491Val variant combination. In agreement with the functional data in zebrafish, the affected individual with p.Ala327Val/p.Phe734Ser variants has milder symptoms excluding developmental delay, intellectual disability, dysmorphic features, abnormal MRI, abnormal neurologic examination, and hearing or vision deficits. Interestingly, the p.Trp220Cys/p.Asp491Val variants showed mild rescue of head but not eye size, again in agreement with the clinical phenotypes of the p.Trp220Cys/p.Asp491Val affected individual who showed retinopathy. Together, our model permitted functional rescue experiments, allowing us to reclassify the majority of known *OGDHL* variants and dissect the specific contributions of these patient variants to disease phenotypes.

## Discussion

*OGDHL* encodes a rate-limiting Krebs cycle enzyme that is proposed to be associated with a highly variable manifestation of neurological and neurodevelopmental disorders including developmental delay/intellectual disability, movement disorders, seizures, dysmorphism, as well as hearing and vision impairment. Notably, many of the clinical features of the patients in our cohort have variably been reported in individuals with biallelic variants in genes encoding enzymes or subunits of enzyme complexes of the TCA cycle. This includes, on the rarer end of the phenotypic spectrum, hypertrophic cardiomyopathy, which is observed in individuals with biallelic variants in *DLD*, *SDHA*, *SDHB,* and *SDHD* (Additional file 6: Table S5) [56]. In this study, we applied an unbiased genotype-first approach screening multiple rare genetic disease databases in order to build a cohort of 14 individuals from 12 unrelated families with biallelic *OGDHL* variants and several distinct clinical entities. Our study intended to explore the gene–disease association through phenotyping, genetic analysis, and functional studies using a zebrafish model. In combination with several inconsistencies in the patient clinical data and rescue studies, our work took an unexpected detour to explore and shape possible explanations in the form of three hypotheses to make sense of our findings, while providing a renewed appreciation of the complexities of the *OGDHL* gene–disease association.

### Hypothesis 1: Biallelic *OGDHL* variants lead to a monogenic disorder with highly variable and significant phenotypic heterogeneity

Highly variable clinical phenotypes are not uncommon for metabolic disorders in general and monogenic disorders associated with deleterious variants of the TCA cycle, ranging from severe syndromes, such as Leigh syndrome, to asymptomatic presentation, underscoring our lack of understanding regarding complexities such as tissue-specific differences in enzyme activity and presence of other genetic and non-genetic factors [57–60]. Here, the homozygous c.2133delA, p.Val712Serfs*77 (individuals 10-11) variant in a healthy sibling while rescuing the zebrafish phenotype, is one potential example of this phenomenon. Furthermore, we deliberately applied a genotype-first approach allowing an unbiased recruitment of patients with biallelic *OGDHL* variants, impartial to phenotype. Upon individual case review, the remarkable presence of variants explaining some, if not most, of the clinical features indicated a significant burden of blended phenotypes, making conclusions about the prevalence of clinical features and discernment of cardinal phenotypes nearly impossible.

There appears to be no clear reason for the discrepancy between the high minor allele frequency and observed phenotype, with certain alleles, such as the previously published c.2201T > C, p.Phe734Ser allele, appearing homozygous in 98 individuals from aggregated variant frequency databases. This also opens the question of hypomorphic alleles, or complex compound inheritance [61], when certain variants only cause disease when *in trans* with specific variants. This was exemplified through compound heterozygous “mimics,” explored through individual and combined variant testing to simulate each scenario. Our work uncovered rescue and failure to rescue with an overall partial rescue when tested together in variants appearing compound heterozygous (e.g., p.Trp220Cys and p.Asp491Val). Nevertheless, the vertebrate zebrafish model allowed a complete variant-testing approach of all previously identified and nearly all newly described variants in an attempt to improve variant reclassification under the presumption that the zebrafish consistently recapitulated the clinical features described in affected individuals. We acknowledge that variability of phenotypes between species is an aspect that cannot be excluded; however, we have nonetheless reclassified the majority of reported variants, despite a large proportion remaining in the VUS categories, eight of which were VUS “favor pathogenic.” The complex findings and reclassifications we report highlight the exceptional challenges of molecular diagnoses for variants in this gene and reflect an extensively broad clinical spectrum associated with all known individuals with *OGDHL* variants to date. Using functional data, we find that variants with hallmarks of pathogenicity as per modified application of ACMG/AMP variant classification guidelines may, in multiple cases, be functionally benign [62–64]. While we used rescue by complementation experiments instead of generating pure genetic mutants for each variant - a limitation of our study - it provides a much faster, adaptable approach to differentiating clearly benign and pathogenic variants. Further investigations will be essential to extend this work and classify the remaining VUS, as some of these may be genuinely pathogenic or benign. We note that since our method was not suitable for evaluation of variants with splice effects, such as p.Val488Val, we could not fully explore pathogenicity in rescue assays, as well as evaluation of phenotype rescue with the compound heterozygous p.Phe734Ser variant. Final classification of many *OGDHL* variants will require collaboration between diagnostic and research laboratories, particularly as more individuals with biallelic *OGDHL* variants are identified. Assays with the potential to test clinical variants on a high-throughput scale are critical resources, although not overcoming of all challenges with atypical observations in metabolic genes, such as those outlined in Hypothesis 2.

### Hypothesis 2: Other roles and mechanisms of *OGDHL* under a non-monogenic and complex inheritance rationale

Several observations challenging the gene-disease relationship of *OGDHL* prompted the exploration of other possibilities. The first informative clue was a simple re-evaluation of the clinical features in individuals 1-3 originating from Iran, Egypt, and Sudan sharing the same homozygous c.1259C > T, p.Thr420Met variant. The overtly unrelated phenotypes, although blurred with other potential diagnoses, served as a first hint that our study must have broader considerations. For example, individuals 1-2 both have microcephaly and abnormal neuroimaging studies, while individual 3, with normal cognition, has neither, but rather presents with abnormal skeletal features such as abnormal vertebrae, scoliosis, pectus carinatum, and joint hyperlaxity, possibly explained by a homozygous *GALNS* (c.1158-3C > G) variant. Furthermore, the significant phenotypic heterogeneity, including an extreme number of disease entities without a cardinal set of features, as well as the observation that many of the individuals we presented had other variants at least partially providing a diagnosis speaks against *OGDHL* as a classical Mendelian disease gene. The family constellations, in many instances, were not supportive for extensive segregation analysis and when possible, it uncovered a peculiar instance of discordant segregation of a homozygous frameshift variant in a healthy individual. In these contexts, we cannot exclude the concept of low penetrance, modifiers impacting age of onset, clinical course, and severity, as well as digenic inheritance with an unknown second gene, environmental effects or that *OGDHL* variants exhibit behavior akin to susceptibility or risk factors. Remarkably, the high minor allele frequency and presence of homozygous individuals in population databases for six variants suggests complex penetrance and expressivity of *OGDHL* variants, if contributing to a monogenic disorder [6]. High conservation of affected amino acid residues did not correspond clearly to pathogenicity of variants as determined by rescue experiments, complicating in silico interpretation. In addition to the important implications of our work for clinical genetics, we report detailed investigation of OGDHL-related proteins and their regulatory interplay. Biological systems possess robustness, a fundamental characteristic that enables maintenance of homeostasis despite deleterious perturbations. Genetic compensation is one mechanism contributing to this robustness, leveraging homologous genes or functionally redundant pathways. Cellular metabolism relies on robust metabolic networks that incorporate backup reactions and alternative pathways to mitigate disruption [65]. For example, previous studies demonstrated that OGDH can partially compensate for loss of DHTKD1 by metabolizing its substrate [9]. Comparatively, individuals with biallelic variants in either *OGDHL* or *DHTKD1* exhibit a very broad clinical presentation, and neither *Dhtkd1* nor *Ogdhl* knockout mice show severe phenotypes, supporting some degree of functional compensation at play [11, 66–68]. Building on this principle, our rescue experiments demonstrate that overexpression of *OGDH* effectively rescues phenotypes resulting from the loss of Ogdhl or Dhtkd1, while the ability of *OGDHL* to rescue Ogdh loss is much more limited. Additionally, biallelic nonsense variants leading to lethality are observed only in individuals with variants in *OGDH* [42], not *OGDHL* [6]. These results suggest that OGDH plays an indispensable role in the regulation of development and can uniquely compensate for the loss of related proteins. Mechanistically, we found evidence of transcriptional compensation in *OGDHL*-related mutants. We observed strong upregulation of *dhtkd1* in zebrafish *ogdhl*-/- mutants, and knockout of the master compensation regulator Upf3a in *ogdhl*-/- mutants reduces this *dhtkd1* upregulation and restores diseased-associated phenotypes. However, contrary to a previous report [37, 38], we did not observe a drastic reduction in expression of compensated genes. This discrepancy may explain the milder phenotypes observed in *ogdhl*-/-;*upf3a* F_0_ compared to *ogdhl* F_0_ animals (stable mutant vs transient F_0_ knockouts). Further investigation is necessary to determine whether simultaneous inhibition of Upf1, Upf3a, and Upf3b is required for complete suppression of genetic compensation, as this is still under debate [69, 70]. Additionally, it is worth exploring whether genetic compensation occurs in *Dhtkd1*/*dhtkd1* stable mutants, and whether overexpression of *DHTKD1* can rescue the phenotypes resulting from the loss of *OGDHL*.

A crucial conclusion from this study, however, is that investigating clinically relevant phenotypes using stable mutants will be challenging due to genetic compensation, so researchers should exercise caution when using stable mutants to investigate metabolic genes [71]. Additionally, although morphological phenotypes serve as a valuable metric for assessing rescue efficiency, further research is required to determine the (im)balance of metabolites in individuals carrying *OGDHL* variants. It is important to acknowledge that our study did not encompass an analysis of the dysmorphic features observed in affected individuals within our zebrafish model. Given that previous and current studies have primarily focused on neural tissues [6], it is imperative to expand research efforts to elucidate the function of OGDHL in other organs.

The development of rescue experiments in the zebrafish model as well as our deeper understanding of genetic compensation in this gene family facilitates the exploration of potential therapeutic approaches to address metabolic diseases associated with 2-oxoglutarate or 2-oxoadipate dehydrogenases. Similar approaches have been used to target pathogenic variants in spinal muscular atrophy 1 (*SMA1*), with promising results from an increase in SMA2 protein expression using antisense oligonucleotides or small molecules [72]. Thus, restoring the enzyme activity or increasing gene expression of *OGDH* could potentially alleviate symptoms caused by loss-of-function variants in *OGDHL* or *DHTKD1* and vice versa. Interestingly, OGDH, OGDHL, and DHTKD1 each possess a ThDP binding domain, and thiamine (vitamin B1) administration is an intriguing treatment possibility, as oral thiamine has been used to improve various neurological disorders with minimal adverse reactions [73–76]. Indeed, thiamine treatment has shown positive effects in stabilizing a subset of metabolites in individuals affected by *OGDH* pathogenic variants [42]. Pursuing a deeper understanding of genetic compensation mechanisms, clinical phenotypes, and metabolic networks involving OGDHL, OGDH, and DHTKD1 will thus not only contribute to a deeper understanding of biological robustness but also have implications for disease diagnosis, treatment, and genetic counseling in individuals with variants in these genes.

### Hypothesis 3: *OGDHL* variants may not be associated with any disease, and they may not be causative at all

Balanced discussion of the conclusions of this study should also consider that *OGDHL* also may not be a disease associated gene. We report multiple individuals with the same variant who show different phenotypes. For example, individuals 1-3 with the c.1259C > T, p.Thr420Met variant in common, and individual 13 in this study and previously described individual 4 family 3 [6], each with the c.980C > T, p.Ala327Val have different phenotypes. Individuals 1 and 3 have other variants that may explain the clinical features while it could be reasoned that the causal variant in individual 2 has not yet been identified. Furthermore, the non-neurological and non-neurodevelopmental disorder phenotype reported in individual 3 who presents an ultra-rare variant affecting a highly conserved amino acid residue (c.1259C > T, p.Thr420Met, skeletal dysplasia), suggests that this variant is most likely not pathogenic in combination with the mismatched phenotype. The same discordance between non-neurological/non-neurodevelopmental disorder phenotypes and highly conserved or deleterious variants can be observed in individual 8 (c.2740A > G, p.Thr914Ala, mainly facial dysmorphism and short stature), individuals 10–11 along with a healthy sibling (c.2133delA, p.Val712Serfs*77, deafness), and individual 14 (c.2273G > A, p.Arg758Gln, cardiomyopathy, and congenital heart defects).

Given the high number of affected individuals with variants explaining at least some of their clinical features, it could simply be that many of the unspecific features that were previously attributed to *OGDHL* are due to variants in other genes. A further example can be shown in the clinical presentation of individual 1, who had an additional *SOX10* pathogenic variant (c.1090C > T, p.Gln364Ter) associated with PCWH syndrome significantly contrasts to individual 3 who had multiple bone deformities, normal brain MRI, and a homozygous VUS (c.1158-3C > G) in *GALNS.* Some of these severe clinical features associated with *SOX10* and *GALNS* overlap with the OMIM descriptions. We also identified individual 13, presenting myopathy, explained by a previously published pathogenic variant in *SELENON*. Similarly, the high minor allele frequency and presence of homozygous individuals in population databases could also suggest unappreciated tolerance to variation and lack of association with disease, in which case, application of the ACMG/AMP guidelines for variant classification, as well as functional testing would be inappropriate.

## Conclusions

The identification of 14 individuals from 12 unrelated families with biallelic *OGDHL* variants showed several associations, leading to the formulation of three distinct hypotheses that support and refute gene–disease causality of *OGDHL* and mechanisms of disease. The vertebrate model was particularly informative and allowed bypassing of genetic compensation in the testing of variants. The consideration of gene compensation in phenotype expression needs to be further studied and may extend to how variants in this gene should be classified and whether ACMG/AMP guidelines can or should be applied. Our results emphasize the importance of strong caution in making definitive diagnoses of “*OGDHL*-related disorders.” It is essential to carefully examine individual cases, as the clinical phenotype may be influenced by other molecular genetics and environmental causes, further contributing to the ambiguity surrounding “*OGDHL*-related disorders.” We hope that our report serves as a blueprint for other laboratories studying genes encoding proteins functioning in complex biochemical pathways where compensatory mechanisms and functional redundancy must be considered for patients presenting ambiguous findings.

### Supplementary Information

**Additional file 1:** **Table S1.** List of primer sequences.

**Additional file 2:** **Fig. S1. **Validation of CRISPR-induced indels in *ogdhl* mutant and F_0_ knockout animals. **Fig. S2. **OGDHL protein orthologs alignment. **Fig. S3. **Temporal expression profiles of *ogdh* paralogs and the tissue-specific expression of *OGDHL*/*ogdhl* across species. **Fig. S4. **Genetic compensation in zebrafish *ogdhl* mutant.**Fig. S5. **Knockout *upf3a* in *ogdhl*-/- mutant elicits disease-associated phenotypes and mitigates the expression of compensated gene.** Fig. S6. **Analysis of the *ogdh* members’ expressions in respective F_0_ knockouts, body length and startle behaviors.** Fig. S7. **Analysis of exogenous human *OGDHL* or *OGDH* expression in co-injected embryos. **Fig. S8. **Analysis of cell apoptotic gene expression in embryos lacking Ogdhl. **Fig. S9.** Molecular modeling of OGDHL variants.** Fig. S10. **Analysis of human *OGDHL* variants’ expression in co-injected embryos.

**Additional file 3:** **Table S2.** OGDHL variants identified.

**Additional file 4:** **Table S3.** Other potential candidate genes identified in our cohort.

**Additional file 5:** **Table S4.** Clinical description of each case including previously published cases.

**Additional file 6:** **Table S5.** Enzymes and their associated disorders and mode of inheritance in Tricarboxylic acid cycle (TCA cycle) pathway.

## Data Availability

The clinical exome and genome sequencing data are not publicly accessible due to their origin in clinical diagnostic labs during patient investigations. Sharing this data outside of the service or without IRB approval is not permitted. Human variant data included in this study have been deposited in the Leiden Open Variation Database (LOVD) and available through the following variant accession numbers: 0000839738 (https://databases.lovd.nl/shared/variants/0000839738#00015015) [77], 0000839739 (https://databases.lovd.nl/shared/variants/0000839739#00015015) [78], 0000839740 (https://databases.lovd.nl/shared/variants/0000839740#00015015) [79], 0000839741 (https://databases.lovd.nl/shared/variants/0000839741#00015015) [80], 0000839742 (https://databases.lovd.nl/shared/variants/0000839742#00015015) [81], 0000839743 (https://databases.lovd.nl/shared/variants/0000839743#00015015) [82], 0000839744 (https://databases.lovd.nl/shared/variants/0000839744#00015015) [83], 0000839745 (https://databases.lovd.nl/shared/variants/0000839745#00015015) [84], 0000839746 (https://databases.lovd.nl/shared/variants/0000839746#00015015) [85], 0000839747 (https://databases.lovd.nl/shared/variants/0000839747#00015015) [86], 0000839748 (https://databases.lovd.nl/shared/variants/0000839748#00015015) [87], 0000839749 (https://databases.lovd.nl/shared/variants/0000839749#00015015) [88], 0000936556 (https://databases.lovd.nl/shared/variants/0000936556#00015015) [89], 0000936559 (https://databases.lovd.nl/shared/variants/0000936559#00015015) [90], 0000936561 (https://databases.lovd.nl/shared/variants/0000936561#00015015) [91], 0000936563 (https://databases.lovd.nl/shared/variants/0000936563#00015015) [92], 0000936565 (https://databases.lovd.nl/shared/variants/0000936565#00015015) [93], 0000936567 (https://databases.lovd.nl/shared/variants/0000936567#00015015) [94], 0000936569 (https://databases.lovd.nl/shared/variants/0000936569#00015015) [95], 0000936572 (https://databases.lovd.nl/shared/variants/0000936572#00015015) [96], 0000936574 (https://databases.lovd.nl/shared/variants/0000936574#00015015) [97], 0000936576 (https://databases.lovd.nl/shared/variants/0000936576#00015015) [98], 0000936578 (https://databases.lovd.nl/shared/variants/0000936578#00015015) [99], 0000936585 (https://databases.lovd.nl/shared/variants/0000936585#00015015) [100], 0000936587 (https://databases.lovd.nl/shared/variants/0000936587#00015015) [101]. Plasmids containing *hOGDHL* (Addgene #211,909) [102] and *hOGDH cDNAs* (Addgene #212,010) [103] are available from Addgene repository.

## Abbreviations

TCA: Tricarboxylic acid
KGDHC: α-Ketoglutarate dehydrogenase complex
OGDH: Oxoglutarate dehydrogenase
OGDHC: 2-Oxoglutarate dehydrogenase complex
OGDHL: Oxoglutarate dehydrogenase-like
DHTKD1: Dehydrogenase E1 and transketolase domain-containing protein 1
QSG: Queen Square Genomics
OMIM: Online Mendelian Inheritance in Man
MRI: Magnetic resonance imaging
dpf: Days post-fertilization
ACMG: American College of Medical Genetics and Genomics
AMP: Association for Molecular Pathology
VUS: Variant of uncertain significance
VUS-FP: VUS-favor pathogenic
VUS-FB: VUS-favor benign
AAALAC: Association for Assessment and Accreditation of Laboratory Animal Care
IACUC: Institutional Animal Care Committee
OMRF: Oklahoma Medical Research Foundation
WT: Wild-type
mm: Millimeters
mm/s: Millimeters per second
ThDP: Thiamine diphosphate
PTC: Premature termination codon
TDT: Terminal deoxynucleotidyl transferase
TUNEL: Terminal deoxynucleotidyl transferase biotin-dUTP nick end labeling
CaP: Caudal primary motor neurons
