# Supplementary material for: Evaluating the association of biallelic OGDHL variants with significant phenotypic heterogeneity
Source: Genome Med. 2023 Nov 29;15:102. doi: 10.1186/s13073-023-01258-4 (PMC10688095; doi:10.1186/s13073-023-01258-4)
Supplement: Supplementary file 2 — Additional file 2: Fig. S1. Validation of CRISPR-induced indels in ogdhl mutant and F0 knockout animals. Fig. S2. OGDHL protein orthologs alignment. Fig. S3. Temporal expression profiles of ogdh paralogs and the tissue-specific expression of OGDHL/ogdhl across species. Fig. S4. Genetic compensation in zebrafish ogdhl mutant.Fig. S5. Knockout upf3a in ogdhl-/- mutant elicits disease-associated phenotypes and mitigates the expression of compensated gene. Fig. S6. Analysis of the ogdh members’ expressions in respective F0 knockouts, body length and startle behaviors. Fig. S7. Analysis of exogenous human OGDHL or OGDH expression in co-injected embryos. Fig. S8. Analysis of cell apoptotic gene expression in embryos lacking Ogdhl. Fig. S9. Molecular modeling of OGDHL variants. Fig. S10. Analysis of human OGDHL variants’ expression in co-injected embryos. [file 13073_2023_1258_MOESM2_ESM.pdf]

Additional file 2

Supplementary Results ... 2
Genetic compensation in zebrafish *ogdhl* mutant ... 2
OGDH variant molecular modeling ... 4
Supplemental Case Reports ... 5
Family 1 ... 5
Family 2 ... 7
Family 3 ... 9
Family 4 ... 9
Family 5 ... 10
Family 6 ... 12
Family 7 ... 14
Family 8 ... 16
Family 9 ... 17
Family 10 ... 17
Family 11 ... 19
Family 12 ... 19
Supplementary Figures ... 20
Fig. S1 ... 20
Fig. S3 ... 24
Fig. S4 ... 26
Fig. S5 ... 28
Fig. S6 ... 30
Fig. S7 ... 31
Fig. S8 ... 32
Fig. S9 ... 33
Fig. S10 ... 34
References ... 35

Supplementary Results

Genetic compensation in zebrafish *ogdhl* mutant

OGDHL/Ogdhl and OGDH/Ogdh display significant protein homology both at the level of protein sequence (Fig. S2E) and 3D structure [1]. These similarities suggest possible functional redundancy [2, 3]. Furthermore, we observed a decrease in *ogdhb* and *dhtkd1* expression coinciding with the onset of *ogdhl* expression at 48 hpf (Fig. S3A), implying possible homeostatic buffering. This hypothesis is supported by the fact that *ogdhl* homozygous null (*ogdhl*-/-) mutants do not exhibit alterations in head size and body length, with only a slight decrease in eye size compared to WT (*ogdhl*+/+) or heterozygous (*ogdhl*+/-) siblings (Fig. S4A). Moreover, *ogdhl*-/- animals survive until adulthood and maintain normal fertility. These findings suggest that any phenotypic defects owing to the lack of OGDHL are ameliorated through genetic compensation and/or redundancy [4]. Furthermore, the absence of significant phenotypes in adult-stage mice with homozygous knockout of *Ogdhl* [5] further supports a similar compensation mechanism in mice [6].

To investigate whether genetic compensation by transcriptional adaptation (increased paralog transcription levels) [6, 7] occurs in *ogdhl* mutants, we examined the mRNA expression of *ogdha, ogdhb*, *ogdhl* and *dhtkd1* using RT-qPCR. Interestingly, we observed a clear upregulation of *ogdhb* and even more strongly *dhtkd1* levels in both *ogdhl*+/- and *ogdhl*-/- embryos at 3 dpf (Fig. S4B). The transcriptional adaptation in the *ogdhl* mutant is consistent with previous reports of compensated genes being upregulated in heterozygous mutants but to a lesser extent than in homozygous mutants [6]. Moreover, *dhtkd1* exhibited persistent upregulation in the *ogdhl*-/- larvae at 6 dpf (Fig.

S4C), whereas upregulated *ogdhb* was only found in *ogdhl*-/- larvae at 3 dpf, suggesting differential involvement of *ogdhb* and *dhtkd1* in genetic homeostasis upon loss of Ogdhl. Previous studies have highlighted the crucial role of Upf1 [6] and Upf3a [7] as essential mediators in triggering genetic compensation responses. Inhibition of either Upf1 or Upf3a in genetic mutants exhibiting compensation can uncover mutant phenotypes similar to those observed in affected humans. However, *upf1* mutants display heart edema and do not survive to 10 dpf [8], while *upf3a* mutants are viable, fertile and exhibit a relatively normal phenotype [7]. To investigate whether genetic compensation was in fact triggered in *ogdhl*-/- animals, we employed a CRISPR/Cas9-mediated knockout strategy to induce bi-allelic mutations in *upf3a* in zebrafish at the founder generation (henceforth referred to as F_0 knockout or F_0). We generated *upf3a* F_0 knockout in WT (*upf3a* F_0) and *ogdhl*-/- (*ogdhl*-/-;*upf3a* F_0) animals and then measured head, eye and body size. Our results revealed a significant reduction in head size and body length in *ogdhl*-/-;*upf3a* F_0 animals compared to *ogdhl*-/- animals at 3 dpf (Fig. S5A-D). Moreover, the smaller eye phenotype was more pronounced in *ogdhl*-/-;*upf3a* F_0 compared to *ogdhl*-/- animals (Fig. S5C). These zebrafish phenotypes resemble clinical symptoms of our patient cohort such as microcephaly and short stature, which are frequently observed in individuals with biallelic variants in *OGDHL*. Furthermore, we observed a significant, albeit incomplete reduction in *dhtkd1* expression in *ogdhl*-/-;*upf3a* F_0 compared to *ogdhl*-/- animals (Fig. S5E). In contrast, the decrease in *ogdhb* expression in *ogdhl*-/-;*upf3a* F_0 animals was not statistically significant (Fig. S5F), suggesting only partial inhibition of genetic compensation by *upf3a* knockout. Taken together, our findings indicate that both *ogdhb* and a metabolically related gene, *dhtkd1*, are upregulated upon *OGDHL* knockout by

genetic compensation mechanisms, resulting in mild to no phenotypes in *Ogdhl* mutant zebrafish or mice, complicating interpretation of gene function *in vivo*.

OGDH variant molecular modeling

Having characterized the role of OGDHL and related genes in neurodevelopmental phenotypes, we next aimed to better understand the specific effects of the variants identified in our cohort. Using homology modeling, we built a structural model of human ODGHL using the recently solved cryo-EM structure of OGDH (1023 aa) and the AlphaFold model for OGDHL (1010 aa), relying on the 75.7% sequence identity of the two proteins, including conservation of the key active site and co-factor binding site residues (Fig. S9). Due to absence of EM coordinates and lower AlphaFold confidence at the extreme N terminus, the structural models were built for residues 129-1010, which encompasses all identified patient variants.

The three novel variants identified in this study are p.(Pro361Ser), p.(Thr914Ala) and p.(Val712SerfsTer77). The p.(Pro361Ser) variant affects a conserved proline located in the α/β1 domain close to the TPP binding site. A change in this residue is predicted to change the protein backbone conformation and affect the cofactor binding site nearby. The variant p.(Thr914Ala) is in the α/β3 domain and is also conserved between OGDH and OGDHL. The model shows a cross-strand Thr914 sidechain to Ile888 backbone interaction, raising the possibility that the Ala substitution abrogates this stabilizing interaction in the β-sheet. The p.(Val712SerfsTer77) variant is predicted to be a Val712 substitution for Ser with further premature termination at position 789. It is likely that transcripts produced from this variant are degraded through the nonsense mediated mRNA decay pathway leading to a reduction in protein level [9].

Other, non-unique variants identified in our patient population include p.(Ser445Leu), p.(Arg496Cys), and p.(Arg869Gln). Ser445 is close to the Ca^{2+} and the Mg^{2+}-TPP binding site, which exhibits negative electrostatic potential, so this change to a nonpolar residue likely has a local effect on the substrate and cofactor binding site. Arg496 is close to the pyrophosphate group of TPP and contributes to the positive electrostatic potential that facilitates stabilization of the negative charge of phosphates along with Mg^{2+} ions. Replacement of Arg with Cys will also affect the TPP binding site, again affecting catalysis. The residue Arg869 is on the surface of the protein and contributes to positive electrostatic potential presumed to contribute to the "tensegrity" of the multimeric E1-E2-E3 complex; thus variants that disturb this surface potential are thought to disrupt the integrity of the complex [10]. Additional, previously reported variants identified in the current study include p.(Trp220Cys), p.(Arg244Trp), p.(Arg299Gly), p.(Ala327Val), p.(Asp491Val), p.(Cys553Leufs*16), p.(Arg673Gln), p.(Phe734Ser), p.(Ser778Leu), p.(Pro852Ala) [11]. These mutations are similarly hypothesized to affect the substrate/cofactor binding site or to affect local interactions.

Supplemental Case Reports

Family 1

Individual 1 is a 3-year-old male child born to first degree consanguineous Iranian parents. He was born at term and had unremarkable prenatal and neonatal histories. His birth measurements were as follows: birth weight 3,700 g, length 50 cm and head circumference 35 cm. He had hypotonia during infancy and severe developmental delay since early life; he was unable to sit or produce words and had severe intellectual

disability. Current growth measurements revealed microcephaly with a head circumference measuring 46 cm (2nd percentile, *-2.2 SD),* short stature with a height of 85 cm (< 1st percentile, *-23.6 SD)*, and failure to thrive with body weight of 9.5 kg (< 1st percentile, *-3.5 SD).* Behavioral problems with stereotypy and temper tantrum were noticed. On examination he was hypotonic and had muscle weakness and atrophy. In addition, he had dystonia, slow dyskinetic movements, alacrimia and bruxism. Auditory assessment revealed severe bilateral congenital non-progressive sensorineural hearing loss. Eye examination revealed albinoid eye with no other prominent dysmorphic features. He was non-ambulatory with no control of bladder and bowel. On general examination he had dextrocardia. Past medical history included operated megacolon and operated Hirschsprung disease, feeding difficulties, constipation, and recurrent urinary tract infections. Brain imaging at the age of 9 months showed mildly prominent subarachnoid spaces overlying the bilateral frontal and temporal lobes, mildly prominent lateral and third ventricles, hypoplastic corpus callosum, anterior commissure, olfactory bulbs, and olfactory sulci, asymmetrically rotated thalami, mega cisterna magna, and right-sided positional plagiocephaly.

Exome sequencing (ES) analysis of individual 1 identified a homozygous missense variant in *OGDHL* (NM_018245.3: c.1259C>T, p.(Thr420Met)). The variant is absent in the Iranome and the Greater Middle East (GME) variome database (Additional file 3: Table S2). Sanger sequencing showed both parents were heterozygous and both healthy siblings were homozygous for the reference allele (Fig. 1A). ES also revealed two further pathogenic variants in individual 1 (Additional file 4: Table S3). The first is a homozygous stop-gain variant found in *PDE6H* (NM_006205.3: c.35C>G, p.(Ser12Ter)). The variant

has been reported as pathogenic for the disease retinal cone dystrophy/achromatopsia 6 (MIM: 610024) in ClinVar and the literature [12]. The other candidate is a pathogenic heterozygous stop-gain variant found in *SOX10* (NM_006941.4: c.1090C>T, p.Gln364Ter). The variant is absent in all known population databases and is classified in ClinVar and the literature [13] as pathogenic for PCWH (peripheral demyelinating neuropathy, central dysmyelination, Waardenburg syndrome and Hirschprung disease) syndrome (MIM: 609136). The *SOX10* variant was not found in the parents as well as both healthy siblings, thus confirming this variant as de novo for individual 1.

Family 2

Individual 2 is a three years and five months Egyptian female child. She is the second child of first-degree healthy consanguineous parents. She had an uneventful prenatal and neonatal history except for 24-hour observation for tachypnoea. Her birth weight was 3 kg, length 49 cm and head circumference 33.5 cm. At the age of 3 months, her parents noticed that she was hypotonic and did not follow, therefore, they started early intervention with physiotherapy and stimulation therapy. She had some delay; she was able to raise her head at the age of 8 months and sat unsupported at the age of one year and two months. Later at the age of 2 years, she started to say a few letters and now can only say 2 to 3 single syllable words. Currently, she can sit unsupported, and stands supported for a few minutes. She showed repetitive, autistic behavior and had good eye contact. Neurological evaluation revealed hypotonia with elicited reflexes, normal sensation, and equivocal Babinski. Growth measurements were below normal for her age; her weight was 10 kg (-3.2 SD), height was 86 cm (-2.8 SD), and head circumference was 47 cm (-2.3 SD). Cranio-facial examination showed brachycephaly with several

dysmorphic features including high forehead, receded anterior hairline, prominent supraorbital ridges, sparse lateral eyebrows, sagging columella of nose, prominent broad chin and large low set ears. In addition, she had alternative squint. She had no history of seizures. Cardiovascular and abdominal assessments were unremarkable. Auditory brainstem response (ABR) testing showed normal hearing.

Brain MRI revealed a small arachnoid cyst in the left middle cranial fossa anterior to the temporal pole and extending into the sylvian fissure, mildly prominent anterior interhemispheric fissure and subarachnoid spaces overlying the frontal convexities suggestive of bifrontal parenchymal volume loss, hypoplastic corpus callosum, and T2 hyperintense signal involving the bilateral parieto-occipital subcortical and periatrial white matter. Karyotyping, extended metabolic screening, organic acid in urine, very long chain fatty acid, lactate, ammonia in plasma were normal.

ES analysis revealed the same homozygous missense *OGDHL* variant as detected in two other individuals (individuals 1 and 3) (NM_018245.3: c.1259C>T, p.(Thr420Met)) (Additional file 3: Table S2). Segregation analysis by Sanger sequencing verified both parents as heterozygous while the brother was homozygous for the reference allele (Fig. 1A). In addition to the c.1259C>T *OGDHL* variant, a homozygous missense variant in *UBQLN4* (NM_020131.5: c.594G>T, p.(Gln198His)) was another candidate for individual 2 (Additional file 4: Table S3). *UBQLN4* has been previously implicated in the pathology of amyotrophic lateral sclerosis (ALS) [14, 15] though the association between variants in *UBQLN4* and ALS has not been established. The *UBQLN4* variant successfully segregated in family 2 in an autosomal recessive manner (Fig. 1).

Family 3

Individual 3 is a 10 years and 6 months Sudanese boy born to consanguineous parents with no previous family history of similar condition. The mother had history of recurrent abortions. Growth assessment showed disproportionate short stature. On neurological examination, he had hypotonia in both upper and lower limbs and was unable to walk without assistance. He was able to write but had very weak hand grip. He had a history of occasional temper tantrum. Variable facial dysmorphic features were identified including abnormal facial shape with coarse facial features, thick eyebrows, depressed nasal bridge, and thick lips. In addition, skeletal assessment revealed abnormal vertebrae with scoliosis, pectus carinatum, and joint hyperlaxity. Abdominal examination identified umbilical hernia. He had normal vision and hearing assessments with no history of epilepsy. Radiological assessment showed a picture of dysostosis multiplex and brain MRI was normal at age of seven years.

ES analysis of the proband revealed the same homozygous missense *OGDHL* variant detected in individuals 1 and 2 (NM_018245.3: c.1259C>T, p.(Thr420Met)). DNA samples were not available from parents and other unaffected siblings for segregation analysis (Additional file 3: Table S2 and Fig. 1). A homozygous VUS was identified in *GALNS* (NM_001323544.2:c.1158-3C>G, p.?) (Additional file 4: Table S3). Additionally, the activity of the enzyme galactosamine-6-sulfate sulfatase was in the pathological range.

Family 4

Individuals 4 and 5 are affected Syrian siblings (brother and sister) who have three healthy brothers, and were born to healthy consanguineous parents. They had poor growth and

short stature. Both were hypotonic on examination and had moderate intellectual disability. There was no history of epilepsy.

Homozygosity mapping, as described in Abou Jamra *et al.*, was applied on individuals 4 and 5, alongside three healthy siblings [16] (Fig. 1A). This resulted in the identification of six candidate homozygosity regions. Further ES on individual 5 narrowed the findings to a homozygous missense variant in *OGDHL* (NM_018245.3: c.2606G>A, p.(Arg869Gln)) (Additional file 3: Table S2). Segregation analysis was also conducted by homozygosity mapping and both individuals 4 and 5 were shown to share the same variant while both parents and the three healthy siblings previously tested all heterozygous (Fig. 1A). Another candidate homozygous stop-gain variant detected in both individuals was found in *CCAR2* (NM_021174.6: c.2482C>A, p.(Tyr828Ter)) (Additional file 4: Table S3).

Family 5

Individual 6 is an 11-year-old African American boy born to consanguineous parents (first degree). He was born at or near term by vacuum-assisted vaginal delivery. His birth measurements were 3.2 kg for body weight and 30.48 cm (1st percentile, *-2.37 SD*) for head circumference. He had hypotonia in infancy and showed severe developmental delay thereafter. In terms of motor delay, he sat unsupported at age of one year and was never able to walk. He had speech delay and can currently say 10 words only in addition to non-verbal communication; he can follow single step gestured commands only and can sometimes point to make a request. He had severe to profound intellectual disability and was unable to perform basic daily activities. There were no behavioural problems, and his hearing and vision were normal. Current growth parameters revealed failure to thrive; the height was 127 cm (1st percentile, *-2.41 SD*), body weight was 25.2 kg (2nd percentile,

-2.14 SD), and the head circumference was 49.4 cm (63rd percentile on Nellhaus curve) (<1st percentile, *-2.86 SD*). Neurological examination showed he was non-ambulatory with muscle weakness and spastic paraplegia (cerebral palsy), decreased axial tone with appendicular musculature hypertonia, hyperreflexia in both upper and lower limbs, and elicited non-sustained ankle clonus. In addition, he had tremor, corrected strabismus and frequent significant drooling. Skeletal abnormalities included bilateral hip dysplasia and spinal alignment mild curvature; R paralumbar fullness. Additionally, had a history of chronic constipation with poor weight gain and seizures in childhood. He was seizure free since the age of 4 years, and his EEG showed diffuse slowing and disorganization of background activity with no epileptiform discharges.

Brain MRI at the age of 4 months revealed diffuse white matter volume loss involving the periventricular and subcortical white matter of both cerebral hemispheres with relative sparring of the anterior frontal lobes. Additional findings included mild cerebellar white matter volume loss, ventriculomegaly predominantly involving the lateral (right more than left) and third ventricles, dysgenesis of the corpus callosum with a markedly hypoplastic rostrum, genu, and anterior body and absent posterior body and splenium with secondary radial configuration of the posterior gyri, absent cingulate gyri, and hypoplastic fornices and hippocampi. In addition, hypoplastic anterior commissure, inferior vermian hypoplasia with widening of the foramen of Magendie and an associated prominent retrovermian CSF-filled subarachnoid space, and anteriorly rotated thalami were noted.

Individual 6 underwent ES and presented a homozygous *OGDHL* variant (NM_018245.3: c.1334C>T, p.(Ser445Leu)). The variant had not been reported in numerous population databases (Additional file 3: Table S2). Sanger sequencing had not been performed at

the time to confirm the variant status for the parents (Figure 1A). A VUS heterozygous missense variant of unknown inheritance was also identified via exome sequencing in *CYFIP2* (NM_001037333.3: c.1355A>T, p.(Glu452Val)) (Additional file 4: Table S3). Variants in this gene are linked to the developmental disease and epileptic encephalopathy 65 (MIM: 618008).

Family 6

Individual 7 is a 20-year-old Iranian male born to first-degree consanguineous parents. He had a normal prenatal and neonatal history. He had a history of speech delay; he was able to say a word at age of one and half years and can say two-word sentences now. He had good non-verbal communication and normal motor development. In addition, he had moderate intellectual disability and was only able to perform some simple basic daily activities. He started to show a progressive course of regression at the age of 4 years. There was no history of behavioural problems or hearing impairment. His growth measurements were within normal range for is age; weight was 60 kg, height was 168 cm and head circumference measured 54 cm. Neurological examination revealed hemiparesis with spasticity in both upper and lower limbs, hypertonia and brisk reflexes. Additionally, he had cerebellar signs on examination including dysdiadochokinesis, dysmetria, truncal and limb ataxia, with gait ataxia. The ataxia was first noticed at the age of 7 years, and he now has moderate to severe ataxia in addition to dysarthria and significant drooling. Furthermore, he had tremor, dystonia, dyskinetic movements (more in upper limbs), and limb contractures. There were no prominent dysmorphic features on craniofacial examination. There was a history of two attacks of focal tonic seizure started

at 18-year-old. EEG showed non-specific scattered sharp waves. There was no neuropathy on examination and both EMG and NCS were normal.

Brain MRI at age of 17 years showed diffuse cerebral and cerebellar white matter volume loss with abnormal T2 hyperintense signal and scattered areas of leukomalacia. Cortical atrophy symmetrically involving both cerebral hemispheres was most pronounced along the paramedian aspect of the frontal and parietal lobes. Additional findings included, mild ex vacuo dilation of the lateral ventricles, T2 hyperintense signal involving the brainstem, dysplastic corpus callosum with a hypoplastic rostrum, genu, and anterior body and markedly hypoplastic and flattened posterior body and splenium with secondary radial configuration of the posterior gyri and absent posterior cingulate gyri and hypoplastic fornices. Markedly hypoplastic anterior commissure, cavum septum pellucidum, and hypoplastic olfactory bulbs were also noted.

Individual 7 revealed homozygosity for a missense variant in *OGDHL* (NM_018245.3:c.1081C>T, p.(Pro361Ser)) through ES. The variant was absent from all known population databases except for UK Biobank (Additional file 3: Table S2). Sanger sequencing verified both parents were heterozygous (Fig. 1A). Two other candidate variants, a homozygous missense VUS in *NDUFS1* (NM_005006.7:c.184C>G, p.(Arg62Gly)) and a published pathogenic homozygous missense variant in *EIF2B3* (NM_020365.5:c.674G>A, p.(Arg225Gln)) [17], were also detected (Additional file 4: Table S3). Pathogenic variants in *NDUFS1* have been reported to cause mitochondrial complex I deficiency, nuclear type 5 (MIM: 618226) whereas the *EIF2B3* variant identified is classified as pathogenic in ClinVar and the literature for the disease

leukoencephalopathy with vanishing white matter (MIM: 603896). Sanger sequencing was not conducted to confirm the segregation.

Family 7

Individual 8 is a 3-year and 5 months European-African female born to first-degree relatives. Her mother was 16-year-old with late pregnancy diagnosis and exposure to alcohol and marijuana. The mother had poor prenatal care and she delivered at term with caesarean section. Prenatal ultrasound revealed polycystic kidneys, confirmed postnatally. The delivery was complicated by perinatal distress, neonatal hypertension with non-oliguric renal failure. In addition, she had neonatal jaundice, poor feeding and prominent dysmorphic feature. She was started on HTN treatment since birth and was followed regularly for her chronic kidney disease. At age of 4 months, cardiological examination revealed patent foramen oval and she developed secondary left ventricular hypertrophy (LVH) later on due to hypertension. At age of 12 months, her foster mother noticed that she was developmentally delayed; she started crawling at age of 11 months, cruising at 19 months old and walking with support at 21 months and was able to say 10 words at the age of 22 months. Her development progressed recently with an improvement in her motor and social skills; however, her mother noticed some memory issues. On growth assessment, she was small for age and had short stature; her height was 88.5 cm (Z score -2.52) and she weighed 12.9 kg (Z score -1.41) for a BMI of 16.5. She had a head circumference of 49 cm (25%). There was a suspicion of staring spells at the age of 1 year, however, the EEG was reported as normal. Neurological examination was unremarkable apart from lower tone for age, one café au lait spot on the right anterior thigh and straight spine with no sacral dimple. She had short neck with dysmorphic

features including frontal bossing, arched bushy eyebrows, synophrys, bilateral epicanthal folds, hypertelorism, telecanthus, right eye esotropia, small upturned wide nose, flattened nasal bridge, thin lips (mainly upper lip), long flattened philtrum, protruding tongue with slight macroglossia tongue, flattened widely spaced teeth, downturned mouth corners, small chin, well-formed posteriorly rotated slightly low-set ears, in addition to small hands and feet. Eye examination revealed right eye esotropia. She had a history of adenoidectomy and recurrent ear infection with suspected hearing difficulties on auditory assessment. Investigations showed elevated renal functions. Since neonatal period, she had arterial hypertension resulted in LVH. Additionally, there was a history of recurrent urinary tract and ear infections and adenitis for which she was treated surgically with adenoidectomy and bilateral ear tube placement. The brain MRI was normal, and the CT scan showed fluid within the mastoid and tympanic cavity on the left with no brain anomalies.

Upon analyzing the ES of individual 8, a homozygous missense variant in *OGDHL* was detected (NM_018245.3:c.2740A>G, p.(Thr914Ala)). The variant is absent from known population databases (Additional file 3: Table S2). The variant status for both parents is undetermined (Fig. 1A). A pathogenic heterozygous splicing variant in *ERF* (NM_006494.4: c.21_22+2delAGGT, p.?) was also found in individual 8. The variant resides on the last two base pairs of exon 1 and the first two base pairs of intron 2 causing a frameshift and resulting in the loss of a splicing junction (Additional file 4: Table S3). Pathogenic variants in *ERF* have been associated with craniosynostosis 4 (MIM: 600775) and Chitayat syndrome (MIM: 617180). The is a suspected *de novo* variant although not confirmed through parental testing.

Family 8

Individual 9 is a 10-year-old German boy born to non-consanguineous parents. There is a family history of epilepsy with no treatment with antiepileptic drugs in the mother who is 53 years old. She had absence seizures and generalized tonic-clonic seizures with last attack of Grand mal seizures at the age of 38 years. In addition, the mother had gestational diabetes during pregnancy. The prenatal history was unremarkable, and he was born at 38 weeks gestation by caesarean section. The condition started at age of twenty-one months when he started to have repeated falls and muscular hypotonia. Later on, at the age of three years and three months, he was diagnosed with myoclonic-astatic epilepsy and was started on a therapy with valproate until the age of seven years and three months. He was suspected to have a coordination disorder at the age of four years; however, this was not confirmed. He was diagnosed with attention deficit disorder at the age of eight years. He showed normal values in the Kaufman-Assessment Battery for Children (K-ABC II). On examination, he had normal growth measurements and there were no dysmorphic features apart from an accessory mammilla. He had normal vision and hearing. A brain MRI was not done.

Trio ES was performed on individual 9 and both parents and compound heterozygous missense variants in *OGDHL* were discovered. The first (NM_018245.3:c.660G>C, p.(Trp220Cys)) was previously reported in a compound heterozygous state in an individual with seizures, dysmorphic features and visual impairment [11] and the second (NM_018245.3:c.1486C>T, p.(Arg496Cys)) that was previously not described (Additional file 3: Table S2). Further ES analysis detected a heterozygous de novo variant in *URB2*

(NM_014777.2:c.1949del, p.(Gly650Valfs*2)) which results in a loss of function (Additional file 4: Table S3).

Family 9

This family was affected with non-syndromic hearing loss, studied and described previously in Doll et al., 2020 [18]. ES of individual 10 identified a homozygous frameshift variant in *OGDHL* (NM_018245.3:c.2133delA, p.(Val712Serfs*77)). The variant was not found in any of the population databases (Additional file 3: Table S2). The same variant was also detected in the affected sibling (Individual 11) and one reportedly healthy sibling (Fig. 1A). Previous exome analysis of individual identified a pathogenic homozygous splicing variant in *CDC14A* (NM_033312.2:c.1421+2T>C, p.(Val472Leufs*20)) (Additional file 4: Table S3) which had been confirmed as pathogenic and segregated within the family. The variant was homozygous only in the two affected individuals with hearing loss and wild type in the unaffected sibling [18].

Family 10

Individual 12 is a 2-year-old Iranian boy born to consanguineous parents. His mother had a history of corrected congenital ASD and VSD postnatally and recurrent first trimester spontaneous abortions. He had severe developmental delay and intractable seizures in the form of generalized tonic-clonic seizures, eye deviation and bouts of apnea started at the age of 4 months. Marked hypotonia was first noticed at age of 12 months and evolved to hypertonia thereafter. He had a history multiple hospital admissions and febrile status epilepticus at the age of 22 months. In addition, his mother reported history of feeding difficulty, but his weight was not affected. His current medications include topiramate, phenobarbitone, levetiracetam, and ketogenic diet. On general assessment, he was

inactive, poorly responsive with no eye contact or social smile, and had no developmental milestones. Additionally, he had plagiocephaly, left torticollis, high arched palate, left cryptorchidism, and a single café au lait macule. Current growth measurements showed DQ <50, HC: 43 cm (-3 to -4 SD), length: 76 cm (-3 to -4 SD). On neurological examination, he had axial hypotonia with extreme head lag with no spontaneous movements, spastic quadriplegia, clenched fists, hyperactive deep tendon reflexes and bilateral Babinski sign. Hearing and vision assessments were normal. EEG at the age of 8 months showed epileptic foci in the left temporal and occipital regions, however, there was no recorded epileptic activity on a sleep EEG at the age of 18 months. Brain MRI at the age of 23 months revealed nonspecific findings, including a thin corpus callosum. Metabolic workup and chromosomal microarray were all normal.

Individual 12 had a paternal cousin with intellectual disability, microcephaly, unilateral hearing loss, poorly understandable speech, behavioural issues, suspected hyperkinetic disorder, hypotonia, left dysplastic kidney and urethral stenosis. His brain MRI revealed frontal polymicrogyria. There is a history of two other paternal cousins, one of them died at age of 20 years, with cognitive impairment with severe intellectual disability, no speech, no walking with significant spasticity. Another distant relative died at the age of 3 years with history of severe developmental delay, acquired microcephaly, failure to thrive, hypertonia and hepatic dysfunction. ES of the proband uncovered a homozygous *OGDHL* variant (NM_018245.3: c.1380C>G p.(Phe460Leu)) (Additional file 3: Table S2) as well as a homozygous likely pathogenic *ADARB1* (NM_015833.4:c.2165C>T, p.Ala722Val) variant (Additional file 4: Table S3) [19].

Family 11

Individual 13 is a 10 year and 7 months old Egyptian boy born to first cousin parents with positive family history of similarly affected deceased twin sister and a cousin. His milestones of development were delayed, he was able to sit at 1 year old and walked at the age of 2 years and 4 months old. Speech was normal, but he had learning difficulty and his IQ was 74 at last exam. On physical examination the weight was 15 kg (-4.12SD), height 136cm (-0.71SD), and head circumference 50cm (-2.29SD). He had weakness in climbing the stairs with positive Gower signs with thin habitus and thin neck. He could elevate the hands above the shoulders and has hypotonia and hyporeflexia. Investigations including creatine phosphokinase (CPK), brain MRI, lactate and pyruvate and cardiac echocardiography were all normal. Exome sequencing of the proband lead to identification of a homozygous *ODGHL* variant (ENST00000374103.9):c.980C>T, p.(Ala327Val) (Additional file 3: Table S2) and an LP homozygous start-loss variant in *SELENON* (NM_020451.2:c.-11_81del, p.(Met1fs)). (Additional file 4: Table S3).

Family 12

Individual 14 is a 4-month-old Iranian girl born to first cousin parents and she has an unaffected older sister. Her growth parameters at birth were within normal range but she was diagnosed with Patent Ductus Arteriosus (PDA) and Hypertrophic Cardiomyopathy (HCM). The surgery was done at the age of 23 days, and she is currently stable with good general heath. Exome sequencing of the proband identified a homozygous *OGDHL* variant (ENST00000374103.9):c.2273G>A, p.(Arg758Gln) (Additional file 3: Table S2) as well as a homozygous VUS in *RAF1* (ENST00000251849.9):c.384A>C, p.(Val128Val) (Additional file 4: Table S3).

Supplementary Figures

Fig. S1

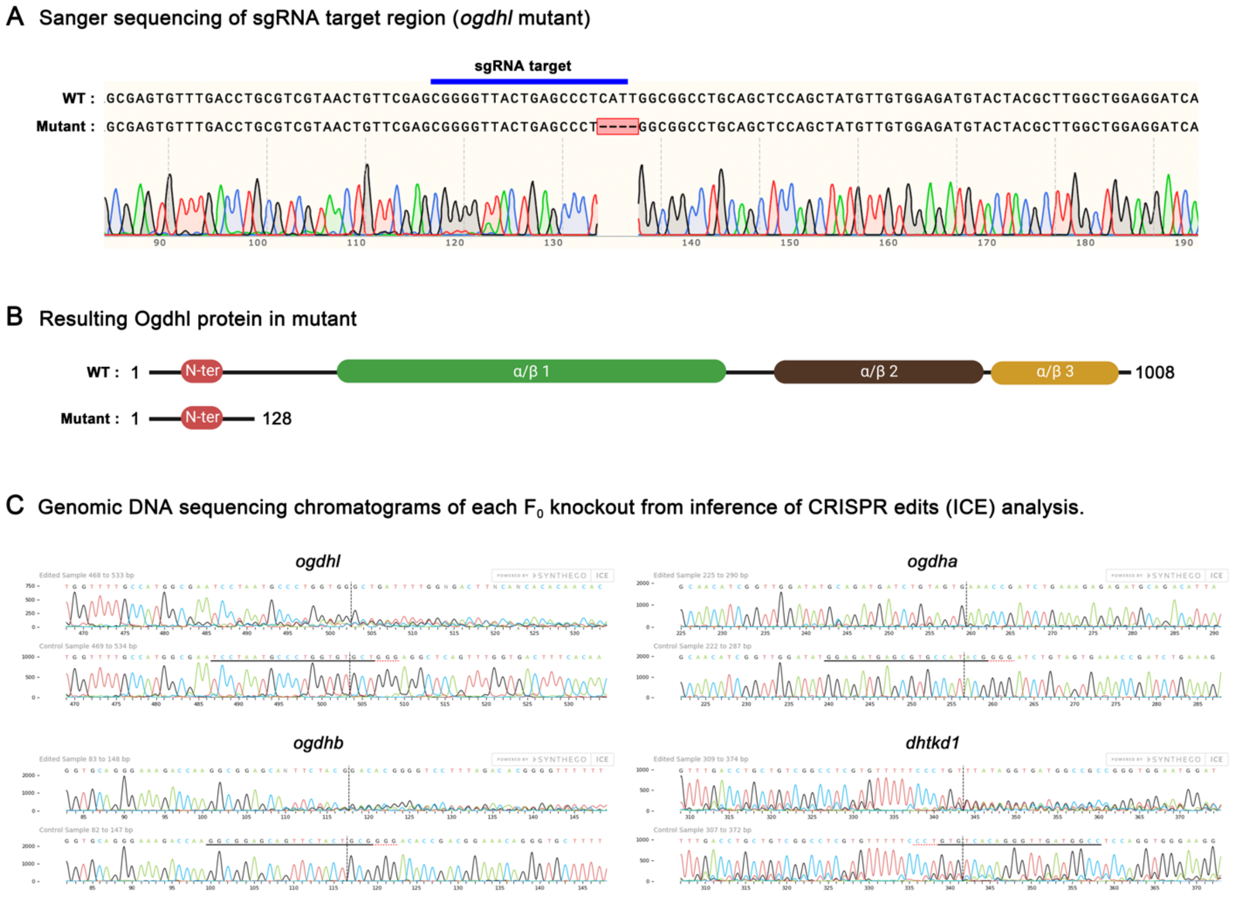

D ICE analysis results

Name	ICE (Indel %)	Indels
ogdhl	87	{'-3': 6.0, '-5': 5.0, '-8': 30.0, '-10': 16.0, '-15': 6.0, '-17': 8.0, '-18': 4.0, '-22': 12.0}
ogdha	98	{'-16': 98.0}
ogdhb	93	{'1': 24.0, '3': 11.0, '-1': 14.0, '-6': 2.0, '-8': 2.0, '-9': 1.0, '-15': 32.0, '-17': 7.0}
dhkd1	87	{'0': 3.0, '4': 1.0, '-3': 35.0, '-8': 28.0, '13': 9.0, '20': 9.0, '-25': 5.0}

Validation of CRISPR-induced indels in *ogdhl* mutant and F_0 knockout animals. A The single-guide RNA target sequence utilized for generating mutant lines of zebrafish *ogdhl*, as well as Sanger sequencing results obtained from the fourth generation of homozygous mutants. The sequencing analysis revealed a 4-base pair deletion in the resulting mutants. **B** The 4-base pair deletion resulted in a premature stop codon, leading to the truncation of the protein (128 amino acids). **C** Genomic DNA Sanger sequencing chromatograms of *ogdhl*, *ogdha*, *ogdhb* and *dhtkd1* F_0 knockouts (lower panel) and their

own controls (upper panel) as analyzed by ICE analysis. Black underline indicates sgRNA target sequence and red dotted underline indicates PAM sequence. **D** Result table showed the ICE score (percentage of indel) for each gene.

Fig. S2

A

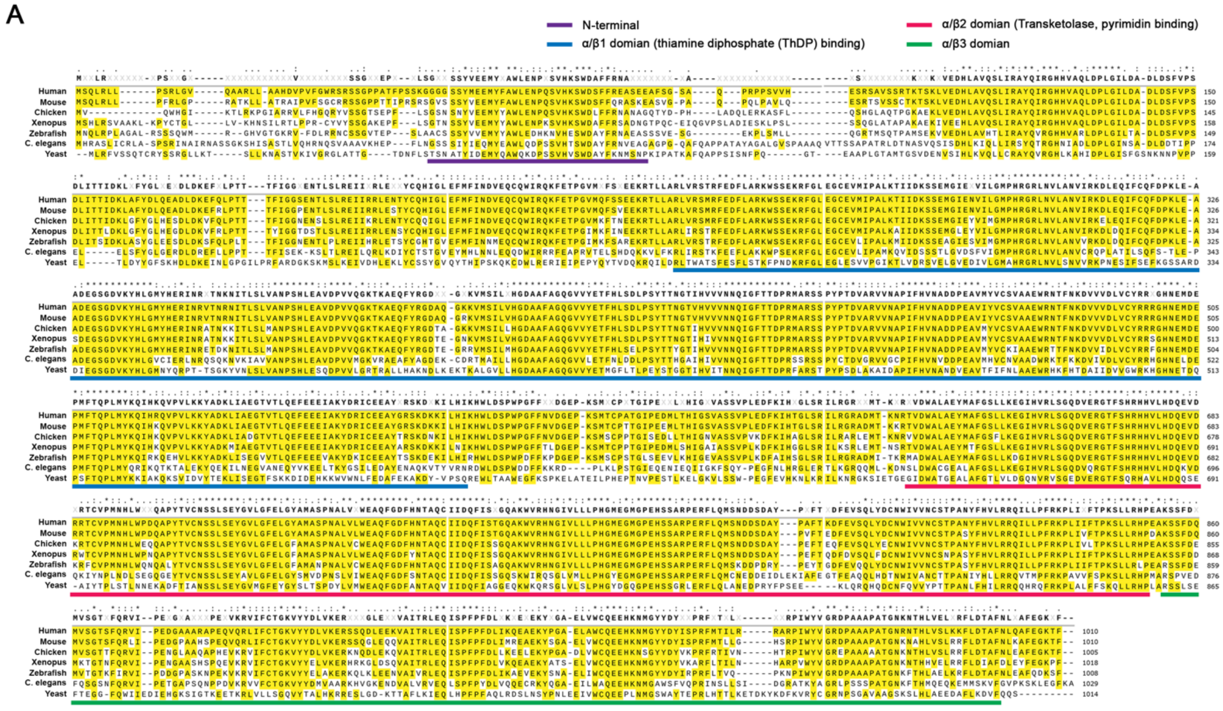

B

Organism	Protein	Identity	Similarity	Gaps
Human	OGDH			
Zebrafish	Ogdha	84.0%	91%	1%
Zebrafish	Ogdhb	82.0%	90%	2%
Human	OGDHL			
Zebrafish	Ogdhl	78.0%	87%	1%
Human	DHTKD1			
Zebrafish	Dhtkd1	71.0%	83%	1%

C

Domain	Region	Indentity
N-terminal	44-78	52%
$\alpha/\beta 1$	250-513	90%
$\alpha/\beta 2$	635-849	91%
$\alpha/\beta 3$	856-998	73%

D

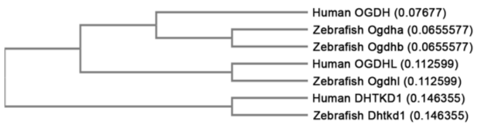

E

Organism	Protein	Identity	Similarity	Gaps
Human	OGDH	100%	100%	0%
Zebrafish	Ogdha	84%	91%	1%
Zebrafish	Ogdhb	82%	90%	2%
Human	OGDHL	77%	87%	2%
Zebrafish	Ogdhl	72%	83%	3%
Human	DHTKD1	37%	53%	13%
Zebrafish	Dhtkd1	36%	54%	13%

OGDHL protein orthologs alignment. A Sequence alignment of human (*Homo sapiens*) OGDHL and corresponding orthologs from mouse (*Mus musculus*), chicken (*Gallus gallus*), xenopus (*Xenopus tropicalis*), zebrafish (*Danio rerio*), C. elegans (*Caenorhabditis elegans*) and yeast (*Saccharomyces cerevisiae*). Highlighted letters on a yellow background represent identical and conserved amino acid residues between species. **B** The homology prediction of OGDH, OGDHL, and DHTKD1 was compared to their orthologs using the DIOPT website (https://www.flyrnai.org/cgi-bin/DRSC_orthologs_v09.pl) including the scores for protein identity and similarity. **C** The protein domain identity of zebrafish Ogdhl compared to human. **D** Phylogenetic tree of

OGDH, OGDHL and DHTKD1 orthologs. **E** The homology prediction of OGDH, OGDHL and DHTKD1 orthologs compared to human OGDH using DIOPT website.

Fig. S3

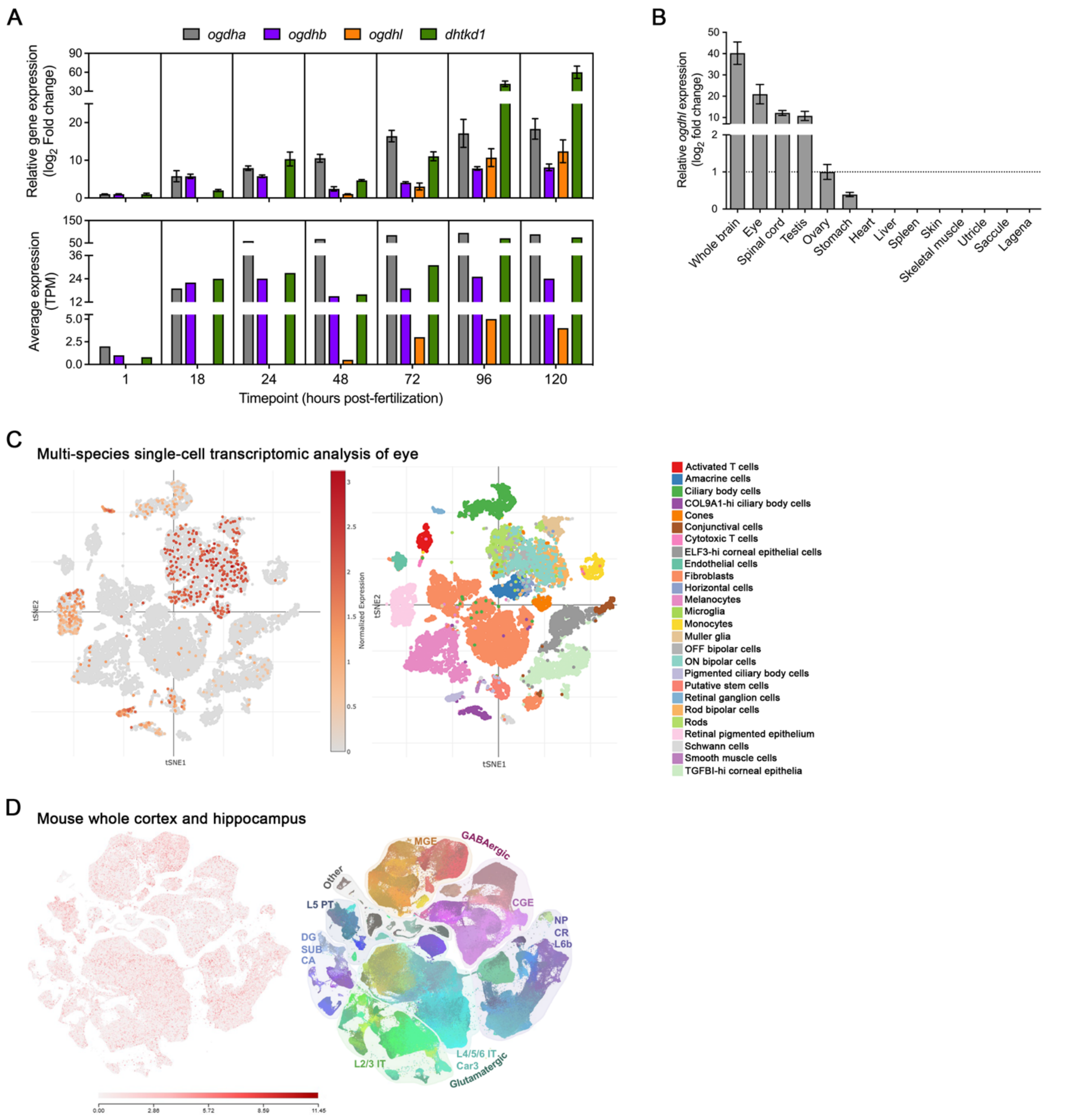

Temporal expression profiles of *ogdh* paralogs and the tissue-specific expression of *OGDHL*/*ogdhl* across species. A mRNA expression of *ogdha*, *ogdhb*, *ogdhl* and *dhtkd1* from 1 to 120 hours post-fertilization (hpf). The upper panel was generated by RT-qPCR and the lower panel was obtained and modified from Zebrafish Expression Atlas (http://www.ebi.ac.uk/gxa/experiments/E-ERAD-475/Results). For RT-qPCR analysis,

each timepoint included biological triplicates as well as technical triplicates. The expression levels were normalized to the housekeeping gene, *18S*.The *ogdha*, *ogdhb* and *dhtkd1* expression levels were compared to 1 hpf stage of embryos, and the *ogdhl* expressions were compared to 48 hpf stage of embryos. **B** RT-qPCR of *ogdhl* mRNA expression in different adult tissues. The expression levels were normalized to *18S* housekeeping gene. Data shown are mean ± SD and compared to ovary. **C** The tSNE plot visualization of *OGDHL* expression in human and porcine ocular compartments (https://singlecell.broadinstitute.org/single_cell/study/SCP1311/multi-species-single-cell-transcriptomic-analysis-of-ocular-compartment-regulons?genes=OGDHL#study-visualize). **D** Visualization of *Ogdhl* expression in the adult mouse cortex and hippocampus using single-cell RNA-seq. *Ogdhl* expression (red) is shown in most transcriptomic cell regions (left). Data were visualized using the Allen Brain Atlas [10x Genomics, https://www.sciencedirect.com/science/article/abs/pii/S0092867421005018?dgcid=rss_sd_all]. Abbreviations for the catalog of transcriptomic cell types: CGE, Caudal ganglionic eminence; CA, cingulate area; CR, Cajal Retzius; DG, dentate gyrus; IT, Intratelencephalically; L2, layer 2; L3, layer 3; L4, layer 4; L5, layer 5; L6, layer 6; L6b, layer 6b; MGE, Medial ganglionic eminence; NP, near-projecting; PT, pyramidal tract; SUB, subiculum.

Fig. S4

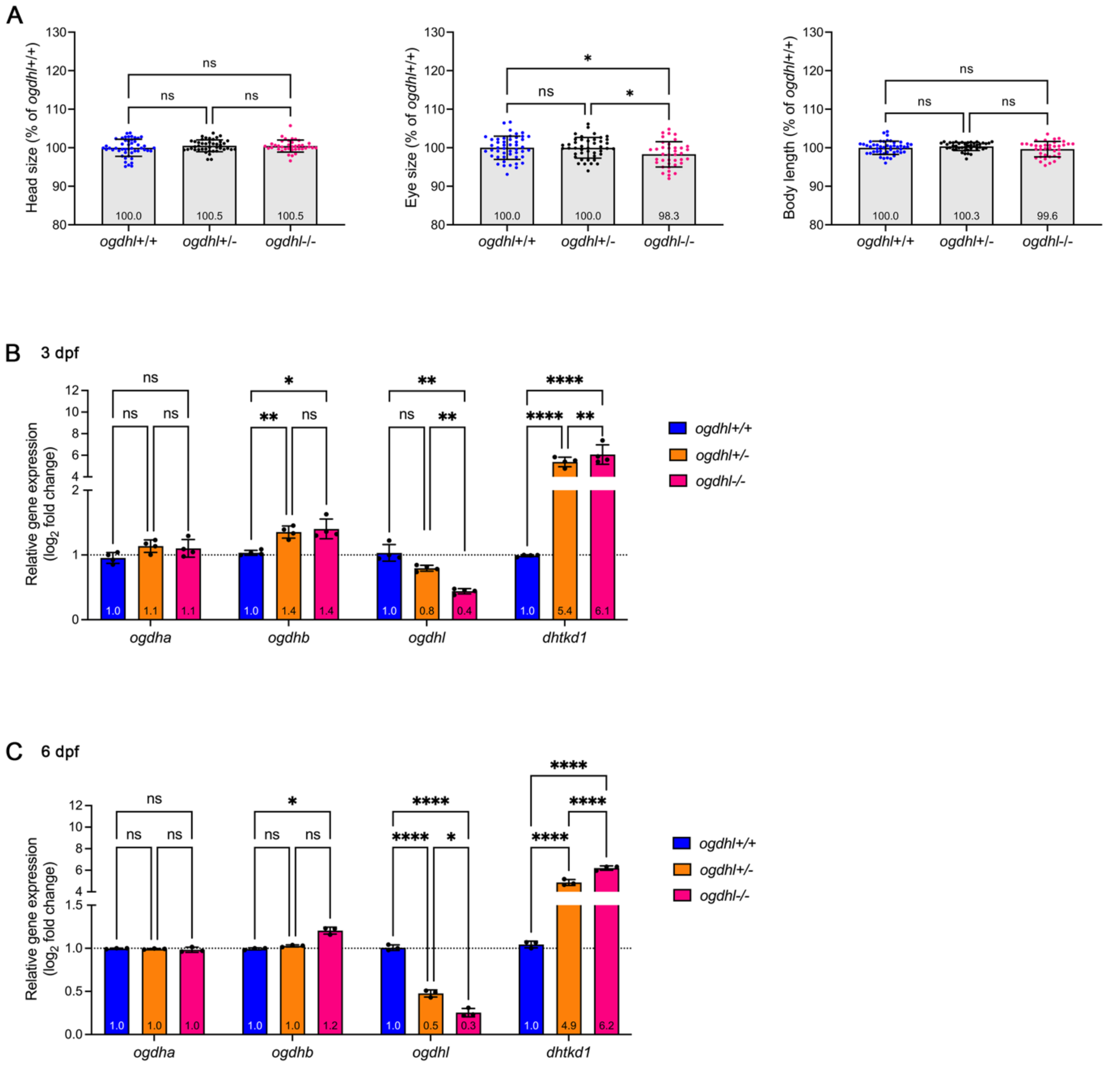

Genetic compensation in zebrafish *ogdhl* mutant. A Size measurements for head, eye and body length in *ogdhl* WT (*ogdhl*+/+, n = 48 embryos), heterozygous (*ogdhl*+/-, n = 43 embryos) and homozygous (*ogdhl*-/-, n = 38 embryos) animals at 3 dpf. All measurements were calculated as a percentage difference compared to the mean value of the WT embryos, and then were compared to each other. Each dot represents one embryo and the mean value of each group was indicated at the bottom of the respective bar in the figure. **B** The RT-qPCR results of *ogdh* paralogs in *ogdhl* mutant animals at 3 dpf. Each group consisted of four biological and technical triplicates. **C** The RT-qPCR

results of *ogdh* paralogs in *ogdhl* mutant animals at 6 dpf. Each group consisted of biological and technical triplicates. For (B, C), the expression levels were initially normalized to the housekeeping gene, *18S*, and then compared to WT embryos to calculate the $\log_2$ fold change. Each dot represents one replicate and the mean value ($\log_2$ fold change) of each group was indicated at the bottom of the respective bar in the figure. Error bars = mean ± SD. Statistical significance was calculated by Brown-Forsthye and Welch's ANOVA with Dunnett's T3 multiple comparisons test: not significant (ns) $p \geq 0.05$, *$p < 0.05$, **$p < 0.01$, ***$p < 0.001$ and ****$p < 0.0001$.

Fig. S5

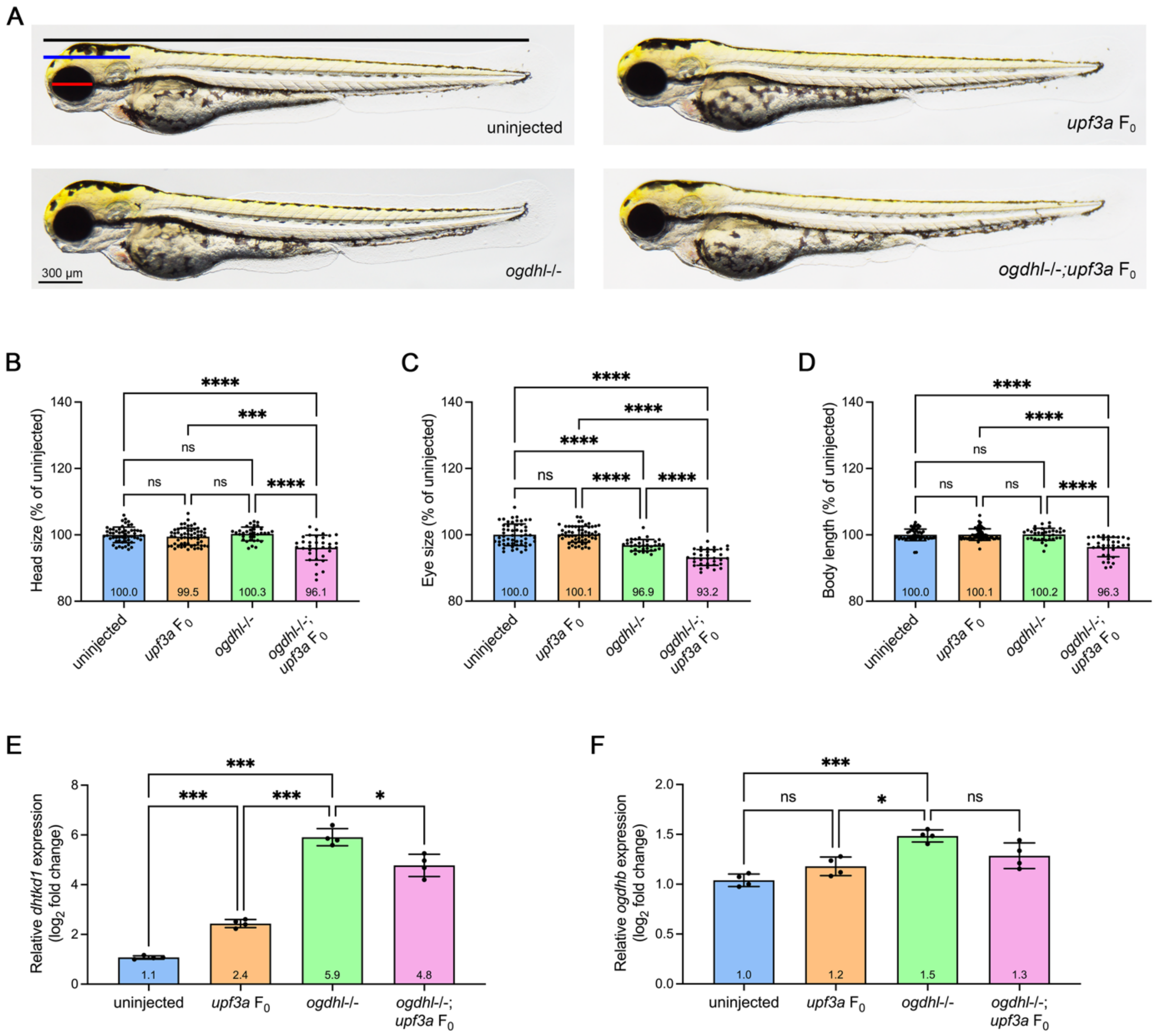

Knockout *upf3a* in *ogdhl-/-* mutant elicits disease-associated phenotypes and mitigates the expression of compensated gene. A Representative image of uninjected, *upf3a* F_0, *ogdhl*-/- and *ogdhl*-/-;*upf3a* F_0 at 3 dpf. **B**-**D** Measurements for head size (blue line), eye size (red line) and body length (black line) as indicated in (A), were calculated as a percentage difference compared to the mean value of the uninjected embryos, and then were compared to each other. Each dot represents one embryo and the mean value of each group was indicated at the bottom of the respective bar in the figure. The number of embryos for uninjected = 55, *upf3a* F_0 = 60, *ogdhl*-/- = 34 and *ogdhl*-/-;*upf3a* F_0 = 34. The RT-qPCR results of **E** *dhtkd1* and **F** *ogdhb* at 3 dpf. Each group consisted of four biological replicates and technical triplicates. The expression levels were

initially normalized to the housekeeping gene, *18S*, and then compared to WT embryos to calculate the $\log_2$ fold change. Each dot represents one replicate and the mean value ($\log_2$ fold change) of each group was indicated at the bottom of the respective bar in the figure. Error bars = mean ± SD. Statistical significance was calculated by Brown-Forsthye and Welch's ANOVA with Dunnett's T3 multiple comparisons test: not significant (ns) $p \geq 0.05$, *$p < 0.05$, ***$p < 0.001$ and ****$p < 0.0001$.

Fig. S6

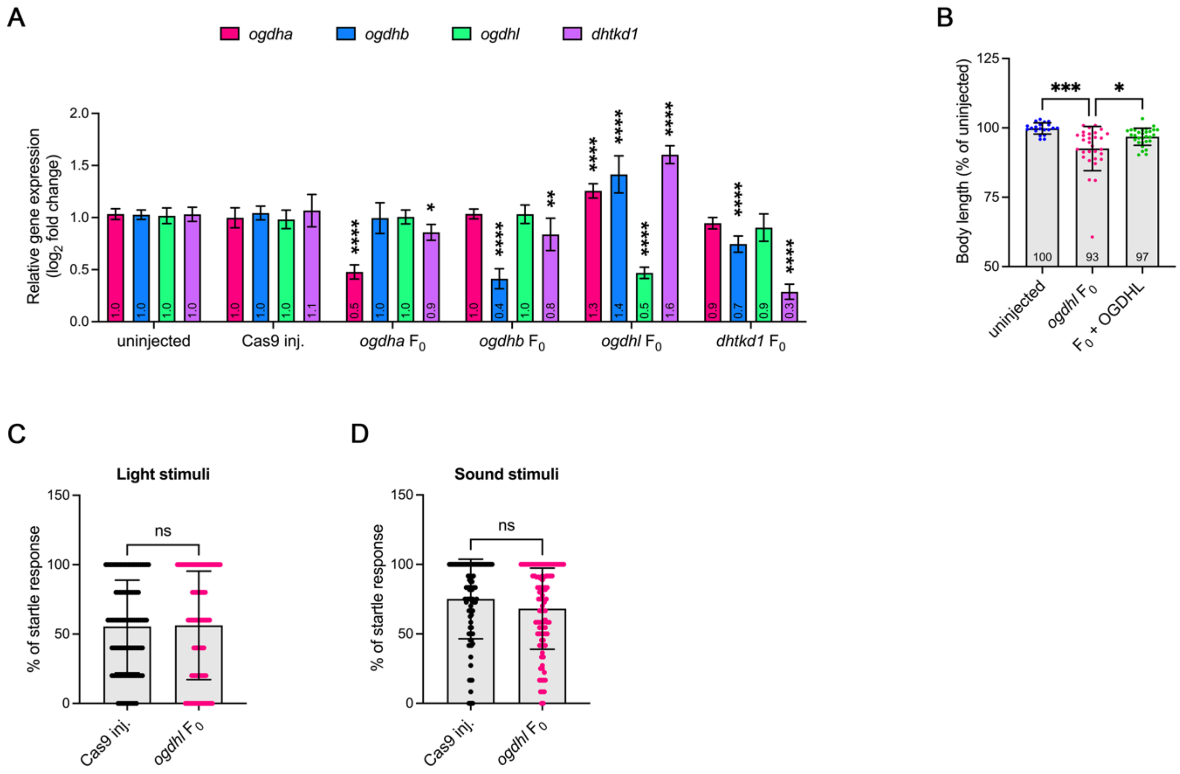

Analysis of the *ogdh* members' expressions in respective F_0 knockouts, body length and startle behaviors. A RT-qPCR results of uninjected, Cas9-injected and F_0 knockout animals (*dhtkd1*, *ogdha*, *ogdhb* and *ogdhl*) at 3 dpf. Each group consisted of four biological replicates and technical triplicates. The expression levels were initially normalized to the housekeeping gene, *18S*, and then compared to uninjected embryos to calculate the $\log_2$ fold change. **B** Body length measurements (black line in A) of uninjected (n = 20 embryos), *ogdhl* F_0 (n = 30 embryos), and F_0 + *OGDHL* (200 pg, n = 30 embryos), were calculated in percentage of difference compared to uninjected embryos. The mean value of each group was indicated at the bottom of the respective bar in the figure. **C** Light stimulation. The number of responses to 5 stimuli of each larva was calculated as a percentage of responses. **D** Sound stimulation. The number of responses to 12 stimuli of each larva was calculated as a percentage of responses. Error bars = mean ± SD. Statistical significance was calculated by Brown-Forsthye and Welch's ANOVA with Dunnett's T3 multiple comparisons test: not significant (ns) $p \geq 0.05$, *$p < 0.05$, **$p < 0.01$ and ****$p < 0.0001$. In (C, D), the Mann-Whitney test was used.

Fig. S7

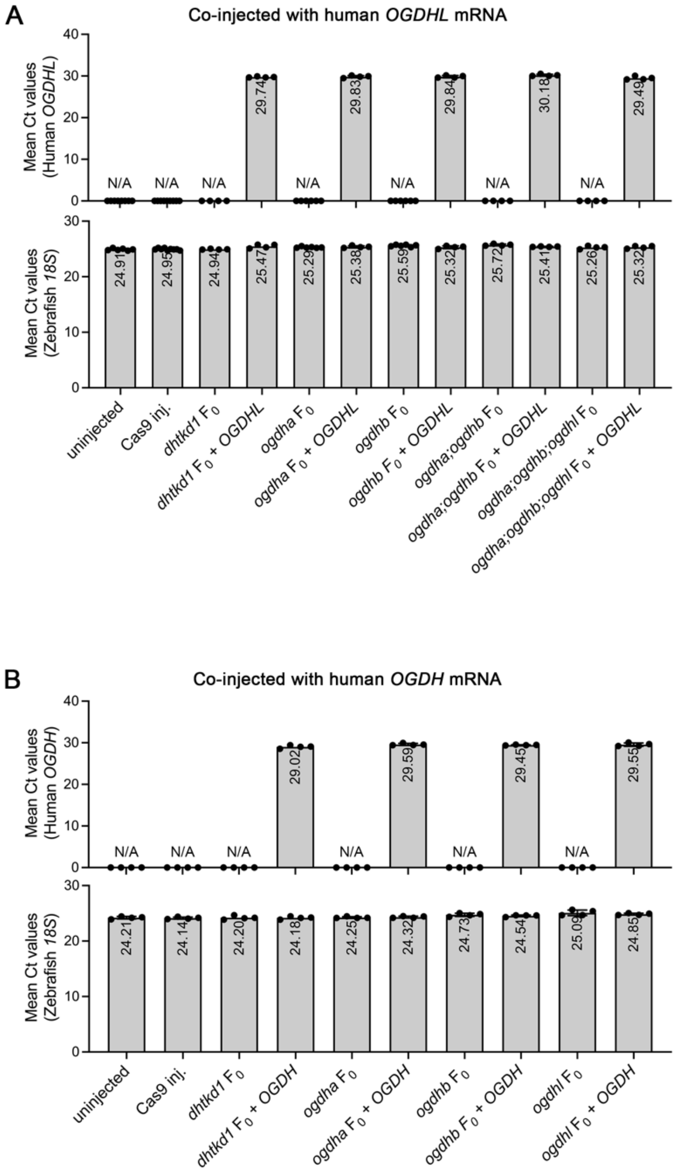

Analysis of exogenous human *OGDHL* or *OGDH* expression in co-injected embryos. RT-qPCR analysis was used to detect the exogenous human *OGDHL* **(A)** or *OGDH* **(B)** mRNA together with *18S* expression in uninjected, Cas9-injected, single knockout (*dhtkd1*, *ogdha* and *ogdhb* F_0), double knockout (*ogdha;ogdhb* F_0) and triple knockout (*ogdha;ogdhb;ogdhl* F_0) embryo at 3 dpf. Each group consisted of four biological replicates and technical triplicates. The mean Ct value of each group was indicated at the top of the respective bar in the figure. Undetectable Ct value is presented as "N/A" (Not Available). Error bars = mean ± SD.

Fig. S8

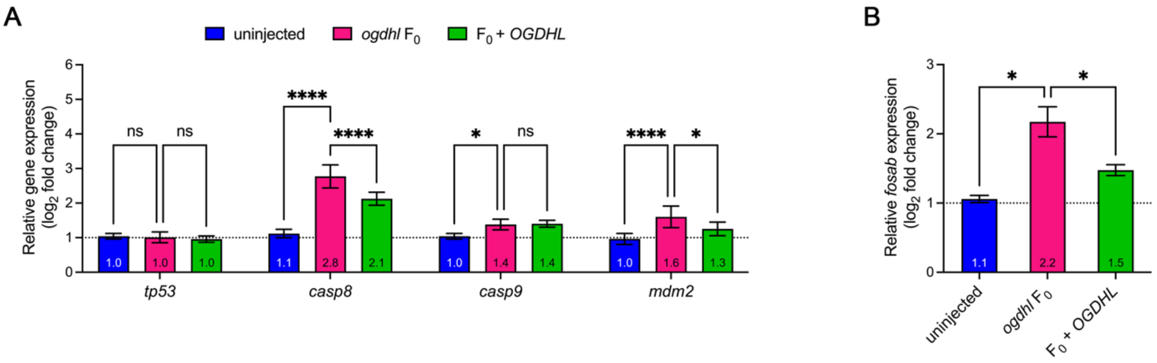

Analysis of cell apoptotic gene expression in embryos lacking Ogdhl. A The results of cell apoptotic gene expression in uninjected, *ogdhl* F_0, and F_0 + *OGDHL* embryos at 3 dpf using RT-qPCR. Each group consisted of four biological replicates and technical triplicates. **B** The results of *fosab* (c-fos) expression in uninjected, *ogdhl* F_0, and F_0 + *OGDHL* embryos at 3 dpf using RT-qPCR. Each group consisted of biological and technical triplicates. The expression levels were initially normalized to the housekeeping gene, *18S*, and then compared to uninjected embryos to calculate the $\log_2$ fold change. The mean value ($\log_2$ fold change) of each group was indicated at the bottom of the respective bar in the figure. Error bars = mean ± SD. Statistical significance was calculated by Brown-Forsthye and Welch's ANOVA with Dunnett's T3 multiple comparisons test: not significant (ns) $p \geq 0.05$, *$p < 0.05$ and ****$p < 0.0001$.

Fig. S9

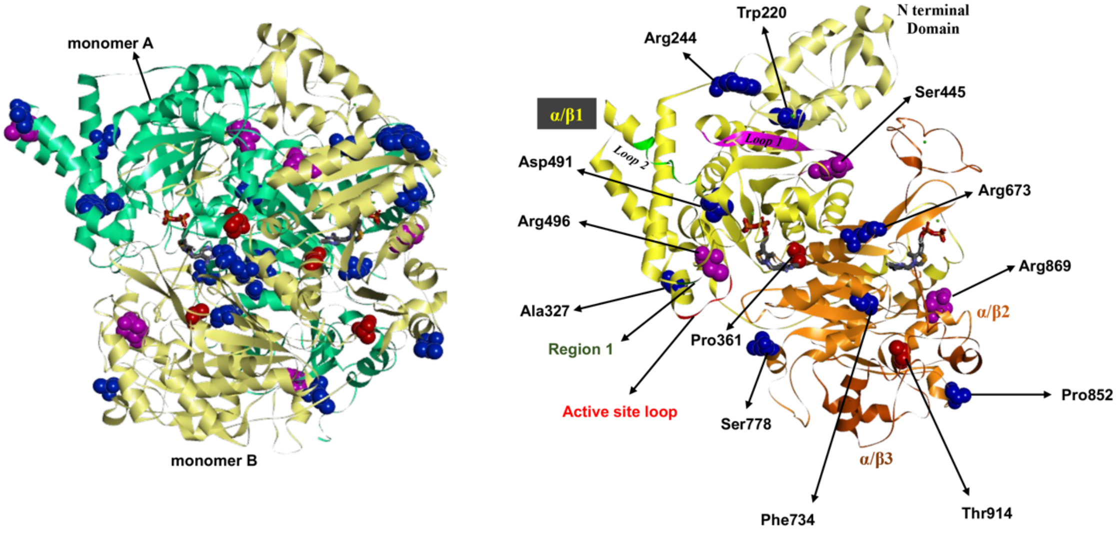

Molecular Modeling of OGDHL variants. Structural model of the OGDHL dimer. Left panel: OGDHL structure is represented in ribbons, monomer A in white, monomer B in sea green. Mg^{2+} and TPP are shown in stick representation and colored green and by atom respectively. Each variant residue is rendered in vDW representation. Variants at position 361 and 914 are colored red, while variants at position 445, 496, 869 are colored pink, while the remaining are colored blue. Right panel-Monomer A is shown alone for clarity and domains are labeled.

Fig. S10

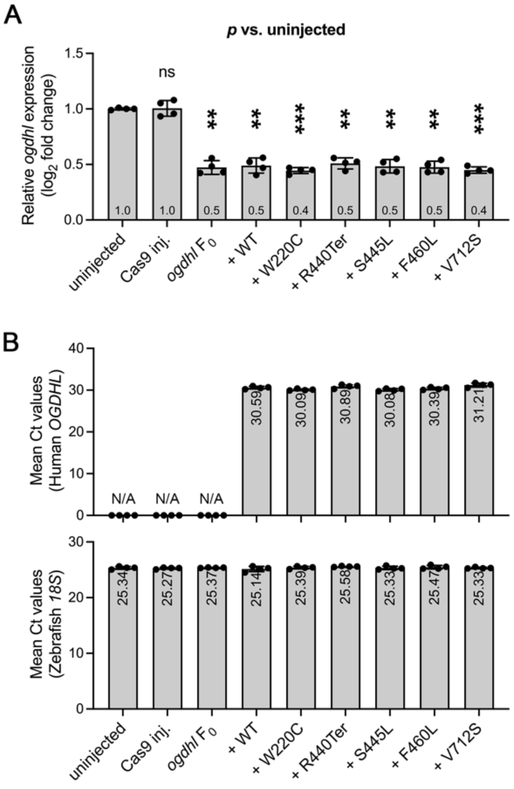

Analysis of human *OGDHL* variants' expression in co-injected embryos. A Endogenous *ogdhl* expression results were obtained from uninjected, Cas9-injected, *ogdhl* F_0 and *ogdhl* F_0 co-injected with various human OGDHL variants at 3 dpf using RT-qPCR. **B** RT-qPCR analysis was used to detect the exogenous human *OGDHL* variant together with *18S* expression. Each group consisted of four biological replicates and technical triplicates. The mean Ct value of each group was indicated at the top of the respective bar in the figure. Undetectable Ct value is presented as "N/A" (Not Available). Error bars = mean ± SD. Statistical significance was calculated by Brown-Forsthye and Welch's ANOVA with Dunnett's T3 multiple comparisons test: not significant (ns) $p \geq 0.05$, $^{**}p < 0.01$ and $^{***}p < 0.001$.

References

1. Bunik VI, Degtyarev D: **Structure-function relationships in the 2-oxo acid dehydrogenase family: substrate-specific signatures and functional predictions for the 2-oxoglutarate dehydrogenase-like proteins.** *Proteins* 2008, **71:**874-890.
2. Singh NP, Krumlauf R: **Diversification and Functional Evolution of HOX Proteins.** *Front Cell Dev Biol* 2022, **10:**798812.
3. Bedner P, Steinhauser C, Theis M: **Functional redundancy and compensation among members of gap junction protein families?** *Biochim Biophys Acta* 2012, **1818:**1971-1984.
4. Rossi A, Kontarakis Z, Gerri C, Nolte H, Holper S, Kruger M, Stainier DY: **Genetic compensation induced by deleterious mutations but not gene knockdowns.** *Nature* 2015, **524:**230-233.
5. Groza T, Gomez FL, Mashhadi HH, Munoz-Fuentes V, Gunes O, Wilson R, Cacheiro P, Frost A, Keskivali-Bond P, Vardal B, et al: **The International Mouse Phenotyping Consortium: comprehensive knockout phenotyping underpinning the study of human disease.** *Nucleic Acids Res* 2023, **51:**D1038-D1045.
6. El-Brolosy MA, Kontarakis Z, Rossi A, Kuenne C, Gunther S, Fukuda N, Kikhi K, Boezio GLM, Takacs CM, Lai SL, et al: **Genetic compensation triggered by mutant mRNA degradation.** *Nature* 2019, **568:**193-197.
7. Ma Z, Zhu P, Shi H, Guo L, Zhang Q, Chen Y, Chen S, Zhang Z, Peng J, Chen J: **PTC-bearing mRNA elicits a genetic compensation response via Upf3a and COMPASS components.** *Nature* 2019, **568:**259-263.
8. Lawir DF, Sikora K, O'Meara CP, Schorpp M, Boehm T: **Pervasive changes of mRNA splicing in upf1-deficient zebrafish identify rpl10a as a regulator of T cell development.** *Proc Natl Acad Sci U S A* 2020, **117:**15799-15808.
9. He F, Jacobson A: **Nonsense-Mediated mRNA Decay: Degradation of Defective Transcripts Is Only Part of the Story.** *Annu Rev Genet* 2015, **49:**339-366.
10. Frank RA, Pratap JV, Pei XY, Perham RN, Luisi BF: **The molecular origins of specificity in the assembly of a multienzyme complex.** *Structure* 2005, **13:**1119-1130.
11. Yap ZY, Efthymiou S, Seiffert S, Vargas Parra K, Lee S, Nasca A, Maroofian R, Schrauwen I, Pendziwiat M, Jung S, et al: **Bi-allelic variants in OGDHL cause a neurodevelopmental spectrum disease featuring epilepsy, hearing loss, visual impairment, and ataxia.** *Am J Hum Genet* 2021, **108:**2368-2384.
12. Kohl S, Coppieters F, Meire F, Schaich S, Roosing S, Brennenstuhl C, Bolz S, van Genderen MM, Riemslag FC, European Retinal Disease C, et al: **A nonsense mutation in PDE6H causes autosomal-recessive incomplete achromatopsia.** *Am J Hum Genet* 2012, **91:**527-532.
13. Inoue K, Khajavi M, Ohyama T, Hirabayashi S, Wilson J, Reggin JD, Mancias P, Butler IJ, Wilkinson MF, Wegner M, Lupski JR: **Molecular mechanism for distinct neurological phenotypes conveyed by allelic truncating mutations.** *Nat Genet* 2004, **36:**361-369.

14. Edens BM, Yan J, Miller N, Deng HX, Siddique T, Ma YC: **A novel ALS-associated variant in UBQLN4 regulates motor axon morphogenesis.** *Elife* 2017, **6**.
15. Senturk M, Lin G, Zuo Z, Mao D, Watson E, Mikos AG, Bellen HJ: **Ubiquilins regulate autophagic flux through mTOR signalling and lysosomal acidification.** *Nat Cell Biol* 2019, **21:**384-396.
16. Abou Jamra R, Wohlfart S, Zweier M, Uebe S, Priebe L, Ekici A, Giesebrecht S, Abboud A, Al Khateeb MA, Fakher M, et al: **Homozygosity mapping in 64 Syrian consanguineous families with non-specific intellectual disability reveals 11 novel loci and high heterogeneity.** *Eur J Hum Genet* 2011, **19:**1161-1166.
17. Ohlenbusch A, Henneke M, Brockmann K, Goerg M, Hanefeld F, Kohlschutter A, Gartner J: **Identification of ten novel mutations in patients with eIF2B-related disorders.** *Hum Mutat* 2005, **25:**411.
18. Doll J, Kolb S, Schnapp L, Rad A, Ruschendorf F, Khan I, Adli A, Hasanzadeh A, Liedtke D, Knaup S, et al: **Novel Loss-of-Function Variants in CDC14A are Associated with Recessive Sensorineural Hearing Loss in Iranian and Pakistani Patients.** *Int J Mol Sci* 2020, **21**.
19. Tan TY, Sedmik J, Fitzgerald MP, Halevy RS, Keegan LP, Helbig I, Basel-Salmon L, Cohen L, Straussberg R, Chung WK, et al: **Bi-allelic ADARB1 Variants Associated with Microcephaly, Intellectual Disability, and Seizures.** *Am J Hum Genet* 2020, **106:**467-483.
